# Work Demands, Burnout, and Withdrawal Behaviors Among Healthcare Workers: A Systematic Review and Meta-Analysis

**DOI:** 10.3390/healthcare14203542

**Published:** 2026-10-21

**Authors:** Ricardo Jorquera-Gutiérrez, Karen Durán-Lara, Emilio Moyano-Díaz

**Affiliations:** 1 Department of Psychology, Universidad de Atacama, Copiapó 1531772, Chile; 2 Faculty of Psychology, Universidad de Talca, Talca 3460000, Chile; karen.duran@utalca.cl (K.D.-L.); emoyano@utalca.cl (E.M.-D.); 3 Instituto de Estudios Humanísticos Juan Ignacio Molina, Talca 3460000, Chile

**Keywords:** work overload, burnout, turnover intention, healthcare workers, meta-analysis

## Abstract

Objectives: This study aimed to determine whether work demands—specifically work overload and mental workload—are associated with burnout and withdrawal behaviors among healthcare workers, and to estimate the magnitude of these associations. Method: A systematic review with meta-analysis and exploratory structural meta-analytic modeling was conducted, following the PRISMA 2020 guidelines. The search covered Web of Science, Scopus, and PubMed/MEDLINE with no restrictions on publication year, yielding 34 studies. Of these, five studies providing six independent samples with comparable data were included in three bivariate random-effects meta-analyses using REML and Fisher’s z-transformation, as well as a two-stage TSSEM to explore the statistical indirect association involving burnout. Results: This review identified heterogeneity in the constructs and instruments used, as well as consistent associations between the variables of interest. The meta-analyses showed positive associations between work overload and burnout, work overload and turnover intention, and burnout and turnover intention. Work overload showed comparatively more homogeneous associations with both burnout and turnover intention, whereas the burnout–turnover intention association showed greater between-study heterogeneity. The exploratory TSSEM showed an indirect association between work overload and turnover intention through burnout, alongside an association between work overload and turnover intention conditional on burnout. Conclusions: Work demands are associated with burnout and withdrawal behaviors. The findings suggest that work overload shows comparatively more consistent associations with burnout and turnover intention, whereas the burnout–turnover intention association appears more sensitive to contextual and measurement-related factors. Burnout may be a relevant correlate within the association between work overload and turnover intention, although longitudinal evidence is needed to establish temporal relationships.

## 1. Introduction

The global health workforce has undergone substantial demographic and occupational changes over recent decades. Between 1990 and 2023, the number of health workers worldwide increased markedly, reaching 122.1 million in 2023, while women accounted for 68.9% of the global health workforce and 71.4% of its net growth. Despite this expansion, substantial differences persist across occupational cadres and regions, alongside important workforce shortages, highlighting ongoing challenges for health workforce planning and retention [1].

Work withdrawal behaviors—including absenteeism, turnover intention, and actual turnover—are important manifestations of employees’ detachment from their work and organization. Absenteeism refers to an employee’s absence from work due to illness or health-related problems [2]. Turnover intention reflects an employee’s conscious intention to leave their job, organization, or profession [3], and may refer to leaving a specific work unit, hospital, or the healthcare profession [2]. In contrast, actual turnover refers to an observed departure from employment and constitutes a distinct behavioral outcome [4]. These distinctions are important because turnover intention should not be equated with actual turnover or broader health workforce shortages.

The Job Demands–Resources model (JD-R) [5,6] defines job demands as physical, cognitive, emotional, or organizational aspects of work that require sustained effort and are therefore associated with physiological or psychological costs. Accordingly, work overload and mental workload are conceptually distinct demands. Work overload refers to a quantitative dimension involving an excess of tasks within a given time frame [7,8], whereas mental workload refers to the cognitive and information-processing effort required by tasks involving concentration, memory, and the simultaneous management of information [9,10]. Although these demands may coexist in healthcare settings, they capture different components of job demands and should not be treated as interchangeable constructs.

Previous research has linked excessive job demands and work overload with burnout and withdrawal-related outcomes among healthcare workers. Work-related fatigue has been linked to absenteeism [7,11,12] and turnover intention [13], whereas burnout has been associated with turnover intention [14,15] and occupational turnover intention [16]. Meta-analytic evidence has also shown an association between burnout and turnover intention among nurses [15], while studies based on the JD-R framework have identified associations between job demands, burnout, and intentions to leave the organization or profession [3,16].

Evidence also suggests that burnout and work-related fatigue may be involved in the association between job demands and withdrawal-related outcomes. Mediation studies have identified indirect associations of job demands with these outcomes through work-related fatigue [13], burnout [17], and, specifically, emotional exhaustion [18]. Longitudinal research further indicates reciprocal relationships between job demands, burnout, and sickness absence over time [19]. However, the predominance of cross-sectional evidence limits causal and temporal interpretations of these relationships.

Despite this growing evidence, significant uncertainties remain. The findings differ depending on the measurement strategy used, with varying results observed between studies that use administrative records and those based on self-reports [20]. Absenteeism has also been operationalized in a variety of ways, ranging from the loss of a single workday to sick leave lasting more than six consecutive weeks. This variability limits comparability across studies and makes it difficult to obtain robust pooled estimates in meta-analyses [20]. Moreover, existing evidence encompasses different healthcare professions, work settings, job-demand indicators, burnout and fatigue measures, and withdrawal outcomes [11,14,15,20,21]. Consequently, although previous research supports associations between job demands, burnout or fatigue, and withdrawal-related outcomes, uncertainty remains regarding which associations can be meaningfully pooled and which require narrative synthesis because of conceptual and methodological heterogeneity.

### 1.1. Problem Statement

Work overload, burnout, and turnover intention represent important concerns for the healthcare workforce. In a recent study of 43,026 healthcare workers from 206 healthcare organizations in the United States, 49.9% reported burnout and 28.7% reported an intention to leave their job [22]. These conditions may affect healthcare workers, managers, patients, and health systems, although turnover intention should not be interpreted as equivalent to actual turnover.

However, differences in definitions, measurement instruments, populations, and withdrawal outcomes limit comparability across studies and determine which relationships can be meaningfully synthesized quantitatively.

### 1.2. Research Purpose

The purpose of this systematic review was to examine the associations between job demands, burnout or work-related fatigue, and work withdrawal outcomes among healthcare workers, and to quantitatively synthesize the associations between work overload, burnout, and turnover intention when sufficiently comparable correlations were available. Narrative synthesis was used to examine associations involving mental workload, work-related fatigue, absenteeism, and actual turnover when study heterogeneity precluded quantitative pooling. Additionally, an exploratory two-stage structural equation modeling (TSSEM) approach was used to examine the pattern of direct and indirect associations between work overload, burnout, and turnover intention. This exploratory analysis was not specified as a priori but was conducted after examining the availability and comparability of the correlations reported in the included studies.

### 1.3. Originality, Rationale, and Significance

Previous reviews have examined burnout, turnover intention, and absenteeism among healthcare workers; however, substantial differences in definitions, measurement approaches, populations, and outcomes continue to limit the comparability and synthesis of the available evidence. Despite this growing literature, a comprehensive study explicitly addressing these methodological boundaries remains needed. The contribution of the present review is to resolve this gap by identifying which relationships can be quantitatively synthesized and which remain too heterogeneous for pooled estimation, while systematically integrating the broader evidence through narrative synthesis.

This approach addresses key theoretical, methodological, and practical necessities in healthcare research. Specifically, this study: (1) establishes boundaries between homogeneous meta-analyses and narrative synthesis; (2) applies exploratory two-stage structural equation modeling (TSSEM) to evaluate the indirect structural association of burnout between work overload and turnover intention; and (3) provides evidence to inform workforce retention strategies in health systems facing severe staffing shortages. Ultimately, these findings contribute to a more precise interpretation of job demands, burnout, and withdrawal behaviors, identifying actionable work-related factors for healthcare management.

### 1.4. Literature Review and Conceptual Framework

The Job Demands–Resources (JD-R) model provides the main conceptual framework for interpreting the relationships between job demands and burnout. The model proposes that job demands require sustained effort and may be associated with physical and psychological costs, whereas job resources may help employees cope with these demands [5,6]. In addition, Conservation of Resources (COR) theory proposes that stress is related to the threat or loss of valued resources [23,24]. Withdrawal and turnover frameworks further distinguish between intention to leave and actual turnover, which represent related but conceptually distinct outcomes [25,26]. Within this conceptual framework, job demands represent the proposed exposure, burnout or work-related fatigue the intermediate constructs, and withdrawal-related behaviors the outcomes of interest.

Empirical evidence among healthcare workers supports some of these associations, although important methodological differences remain. Rotenstein et al. [22], in a large cross-sectional study across healthcare occupations, found associations between work overload, burnout, and intent to leave, although the design precluded temporal inference. Petersen et al. [7] reported an association between emotional exhaustion and self-reported sickness absence among nurses, whereas Van der Heijden et al. [16] longitudinally examined relationships between job demands and resources, burnout, and occupational turnover intention among nurses. More recent longitudinal evidence showed that specific job resources, including job control and collegial support, may buffer some associations between job demands and subsequent burnout complaints among healthcare professionals [27]. Overall, these studies support associations between job demands, burnout, and different withdrawal outcomes, while differences in study design, populations, measurement approaches, and outcomes limit direct comparability.

In the present review, these conceptual frameworks guide the interpretation of the evidence but are not themselves directly tested. The meta-analysis examines three associations for which sufficiently comparable correlations were available: work overload–burnout, work overload–turnover intention, and burnout–turnover intention. Other relationships involving mental workload, work-related fatigue, absenteeism, and actual turnover are examined narratively.

## 2. Materials and Methods

Study Type. This study is a systematic review with meta-analysis and exploratory structural meta-analytic modeling. It synthesizes the available evidence on the relationships between work overload, mental workload, burnout, and withdrawal behaviors among healthcare workers. The review followed the PRISMA 2020 guidelines and transparently addressed eligibility criteria, information sources, search strategy, study selection, data extraction, methodological appraisal, and quantitative and narrative synthesis methods.

The literature search included the Web of Science Core Collection, Scopus, and PubMed/MEDLINE, with no restriction on publication start date; searches were completed in July 2026. Eligible studies were original peer-reviewed articles published in English or Spanish, with no initial restrictions by country or healthcare setting. Studies were required to include healthcare workers and to assess all three predefined domains within the same study: work overload, mental workload, job demands, or equivalent variables; burnout, emotional exhaustion, or work-related fatigue; and intention to leave, actual turnover, absenteeism, sick leave, or presenteeism. Based on the studies ultimately included, the synthesized evidence covered publications from 2007 to 2026; therefore, this period reflects the range of the included studies rather than an a priori defined time window.

Search strategy. The search strategy combined controlled vocabulary and free-text terms related to mental workload, work overload, job demands, burnout, and adverse work outcomes. The base search string was: (“Mental workload” OR “Cognitive load” OR “Work overload” OR “Job demands” OR “workload”) AND (“Burnout” OR “Professional burnout” OR “Emotional exhaustion”) AND (“Absenteeism” OR “Sick leave” OR “Personnel turnover” OR “Turnover intention” OR “Work absence” OR “presenteeism”) AND (“Health Personnel” OR “healthcare worker*” OR “health care worker*” OR “healthcare professional*” OR “health care professional*” OR “healthcare provider*” OR “medical staff” OR “hospital staff” OR “clinical staff” OR “health workforce” OR “physician*” OR “doctor*” OR “clinician*” OR “nurse*”). The syntax was adapted to each database according to available search fields, operators, and filters.

Inclusion and exclusion criteria. The analysis included quantitative observational, longitudinal, cross-sectional, modeling, or quasi-experimental studies with analyzable data; a study population consisting of healthcare workers; explicit measurement of work overload, mental workload, job demands, time pressure, staffing levels, quantitative workload, or equivalent variables; measurements of burnout, emotional exhaustion, work fatigue, or related constructs using validated instruments, standardized scales, or clearly described measures; and reporting of outcomes related to intention to leave, retention intention, actual turnover, absenteeism, or sick leave. The meta-analysis also required that studies provide sufficient data to estimate a comparable statistical association between the variables of interest.

Editorials, letters, commentaries, narrative reviews, qualitative studies without quantitative estimates, studies not involving a healthcare population, studies without defined occupational outcomes, duplicate records, and systematic reviews were excluded. When the same study was reported in more than one publication, the version with the most complete information or the larger sample size was retained.

The selection process was conducted in two phases: first, screening of titles and abstracts; second, review of the full text. Two reviewers (RJG and KDL) independently screened titles and abstracts, reviewed full-text articles, extracted data, and evaluated the risk of bias using the JBI tools. Disagreements at any stage were resolved through discussion or consultation with a third reviewer (EMD). The flowchart for the identification, selection, eligibility, and inclusion of studies is presented in the PRISMA diagram (see Figure 1). For each included study, the following information was collected: author, year, country, study design, population, sample size, healthcare setting, instruments or measures of work overload and mental workload, instruments or measures of burnout or fatigue, type of work-related outcome, association estimators, adjustment variables, and main results. Prior to final selection, four studies were excluded as they did not meet the inclusion criteria (see Table A4). Exclusion decisions were reviewed to ensure consistency with the requirement that all three domains be measured within the same study. Ultimately, 34 studies were included in the systematic review.

The methodological quality of the 34 included studies was assessed using the Joanna Briggs Institute (JBI) critical appraisal tools, selected according to each study’s design. The JBI Critical Appraisal Checklist for Analytical Cross-Sectional Studies was used for analytical cross-sectional studies [28], the JBI Critical Appraisal Checklist for Cohort Studies was used for longitudinal cohort studies [29], and the JBI Critical Appraisal Checklist for Quasi-Experimental Studies was used for quasi-experimental studies [30]. Each criterion was classified as “Yes,” “No,” “Unclear,” or “Not applicable,” as appropriate.

The JBI assessments were used to characterize design-specific methodological limitations and to inform the interpretation of the quantitative synthesis rather than as an automatic criterion for excluding studies from the meta-analysis. Eligibility for quantitative synthesis was determined by conceptual and statistical comparability and the availability of suitable effect-size data. JBI findings were subsequently used to inform the risk-of-bias domain of the GRADE assessment.

Registry. This systematic review was not registered in PROSPERO or any other registry, and a formal protocol was not published prior to conducting the study.

### Data Analysis and Synthesis

Data synthesis was conducted at three complementary levels: narrative synthesis, bivariate meta-analysis, and exploratory two-stage structural equation modeling meta-analysis (TSSEM).

Narrative synthesis. All 34 included studies were examined through a structured narrative synthesis, considering study design, healthcare population, occupational demands, burnout or work-related fatigue measures, and withdrawal-related outcomes. Findings were organized according to the exposure, intermediate construct, and occupational outcome assessed. Studies involving mental workload, work-related fatigue, absenteeism, presenteeism, or actual turnover were synthesized narratively when differences in constructs, measurement instruments, or statistical estimates precluded quantitative pooling.

To assess direction and consistency, a direction-of-effect consistency rule was applied based on the percentage of studies reporting statistically significant associations (*p* < 0.05). Evidence was classified as follows:

Consistent/Conclusive: when ≥75% of the studies examining the association reported a statistically significant association in the same direction.

Mixed/Inconsistent: when between 30% and 74% of the studies reported statistically significant associations in the same direction, or when statistically significant effects in opposite directions were observed across studies.

No evidence of association: when fewer than 30% of the studies reported statistically significant associations. Additionally, measures were stratified according to data source into: (a) self-reported measures and (b) objective or administrative records (administrative/registry data).

Bivariate meta-analysis. Quantitative synthesis was restricted to studies reporting comparable zero-order Pearson correlation coefficients (r) and corresponding sample sizes (N) across the triad of variables of interest. Correlations derived from other statistical estimators, including Spearman coefficients, regression coefficients (β), and odds ratios (OR), were not converted into Pearson correlations. Studies reporting partialized estimators (e.g., SEM path coefficients, regression betas), logistic metrics (odds ratios), non-parametric estimates (Spearman’s rho), or those assessing non-eligible withdrawal outcomes were excluded from the quantitative pooling. A comprehensive description of the methodological criteria and study-by-study justifications for exclusion from the meta-analyses is provided in Supplementary Note S1 and Supplementary Table S1.

Three associations were examined: work overload–burnout, burnout–turnover intention, and work overload–turnover intention. Pearson correlations were transformed using Fisher’s z-transformation, and random-effects models were estimated using restricted maximum likelihood (REML). Pooled estimates were subsequently back-transformed into Pearson correlation coefficients to facilitate interpretation.

Statistical heterogeneity was assessed using Cochran’s Q statistic, I^2^, and τ^2^. Sensitivity analyses were conducted by examining studentized residuals, using Bonferroni-corrected critical thresholds to detect potential outliers, and by reviewing influence diagnostics to identify studies with a disproportionate impact on the pooled estimates. Publication bias was evaluated through visual inspection of funnel plots, Begg’s test, Egger’s test, the trim-and-fill method, and Rosenthal’s fail-safe N. Given the limited number of independent samples, these assessments were interpreted cautiously.

Certainty of evidence. The certainty of evidence for each meta-analytic association was evaluated using the GRADE (Grading of Recommendations Assessment, Development and Evaluation) approach [31]. Because the quantitative synthesis was based on observational studies, the initial certainty of evidence was rated as low. Certainty was then assessed across the GRADE domains of risk of bias, inconsistency, indirectness, imprecision, and publication bias, and potential upgrading criteria were also considered. The risk-of-bias domain was informed by the design-specific methodological limitations identified through the JBI critical appraisal. Inconsistency was evaluated considering the magnitude and statistical significance of heterogeneity, the I^2^ statistic, and the consistency of effect directions and prediction intervals. Imprecision was assessed considering the width of the confidence intervals around the pooled estimates, while indirectness was evaluated in relation to the populations, exposures, and outcomes addressed by the review question. Publication bias was assessed considering funnel-plot asymmetry and statistical tests, while considering the limited number of studies available for each meta-analysis. The rationale for downgrading or maintaining certainty within each domain was documented for each meta-analytic association.

Exploratory structural meta-analytic modeling. The TSSEM was conducted using the pooled zero-order correlation matrix constructed from the bivariate meta-analyses of work overload, burnout, and turnover intention. Structural parameters were estimated via Weighted Least Squares (WLS) using the metaSEM package version 1.5.0 in R. To properly propagate estimation uncertainty from the primary studies into the structural paths without inflating statistical precision, the harmonic mean of primary sample sizes (N_harm_ = 1150) was used as the effective Stage 2 sample size, and asymptotic standard errors and 95% confidence intervals were computed for direct (c), indirect (a X b), and total effects. Complete analytical scripts for reproducibility are provided in Supplementary Note S2. Given the predominance of cross-sectional studies, the aggregated nature of the correlations, and the limited number of independent samples, the TSSEM was considered exploratory and associative, without establishing temporal or causal mediation.

## 3. Results

This section first describes the characteristics of the included studies, followed by a narrative synthesis of the constructs, instruments, and main associations identified in the review. The meta-analytic findings and the structural meta-analytic mediation analysis are then presented.

### 3.1. General Characteristics of the Included Studies

The 34 studies in this systematic review (see Table 1) were published between 2007 and 2026, primarily in Europe and North America, and less frequently in Asia, Oceania, and South America. Studies in nursing and cross-sectional designs predominated.

Sample sizes varied widely, ranging from 60 participants in specialized units [10] to 43,026 in national surveys [22]. Nurses represented the primary study population in most studies (62%), although several studies also included physicians [31,32] or mixed groups of healthcare personnel. Cross-sectional designs predominated (82.4%), followed by longitudinal studies (11.8%) [16,32,33] and quasi-experimental intervention evaluations (5.9%) [34,35].

Evaluation using the Joanna Briggs Institute (JBI) tools [28,29,30] identified specific risks of bias relevant to the interpretation of the findings (Table A1, Table A2 and Table A3). Among cross-sectional studies, the main concerns involved the identification and control of potential confounding factors, with several studies showing unclear or insufficient reporting in these domains (Table A1). Given the cross-sectional nature of these studies, temporal ordering between job demands, burnout or work-related fatigue, and withdrawal-related outcomes could not be established.

Among longitudinal studies, exposure and outcome measurement were generally adequate, and confounding factors were addressed in most studies (Table A2). However, none of the studies required participants to be free of the outcome at baseline, although Adriaenssens et al. [32] and Thapa et al. [33] adjusted for baseline outcome levels. Follow-up was another relevant concern: incomplete follow-up or varying analytical sample sizes were observed in Adriaenssens et al. [32], Thapa et al. [33], and Van der Heijden et al. [16], whereas Cheyroux et al. [36] used full information maximum likelihood to address missing longitudinal data. These limitations warrant caution when interpreting temporal relationships.

Among quasi-experimental studies, the main limitation was the absence of multiple outcome measurements before and after the intervention or exposure in both studies [34,35]. In addition, Rickard et al. [35] did not include a control group, and comparability of care apart from the intervention was unclear (Table A3). These limitations reduce confidence in attributing observed changes specifically to the intervention or exposure.

**Table 1:** Characteristics of the included studies (N = 34): authors, country, data collection period, study design, setting, population, sample, instruments, and results.

Reference	Country/Region	Data Collection Period	Study Design	Setting/Context	Population	Sample (N)	Instruments/Measures	Results
Adriaenssens et al. (2015) [32]	Flanders, Belgium	2007–2009	Panel longitudinal study (two waves)	General hospitals; emergency departments	Emergency department nurses	170	LQWQ-N; UWES; MBI; BSI	Time pressure and workload predicted higher levels of emotional exhaustion and intention to leave the profession.
Albini et al. (2011) [37]	Northern Italy	Unspecified (prior to 2008)	Cross-sectional	Public hospital; internal medicine	Physicians, nurses, and support staff	230	JCQ; STAI; MBI; objective parameters	The patient-to-staff ratio was associated with emotional exhaustion and more days of sick leave.
Bruyneel et al. (2017) [38]	Flanders, Belgium	2014–2015	Multicenter cross-sectional study	Emergency departments	Emergency room nurses	292	LQWQ-N; PES-NWI; MBI-HSS	The work environment influenced job satisfaction, emotional exhaustion, and intention to leave.
Enea et al. (2024) [39]	Belgium, the Netherlands, Italy, and Poland	May–September 2022	Multicenter cross-sectional study	Eight European hospitals	Nurses with a bachelor’s degree	1350	Online METEOR questionnaire: demands, resources, and burnout	Emotional exhaustion and depersonalization were the main predictors of intention to leave.
Gabel Shemueli et al. (2016) [8]	Uruguay and Spain	January–December 2012	Cross-sectional comparative study	Public hospitals	Registered nurses	818	TIS; Shirom–Melamed Burnout; UWES; social support; Karasek	Workload was associated with intention to leave, fully mediated by burnout.
Hämmig (2018) [40]	Switzerland	2015–2016	Cross-sectional	Hospitals and rehabilitation clinics	Doctors, nurses, and other healthcare professionals	1840	CBI; COPSOQ; ERI; work–life imbalance measures	Mental, physical, and emotional stressors were associated with burnout and intention to leave.
Hewko et al. (2015) [41]	Western Canada	2007–2010	Cross-sectional (Phase 2 of QWEST)	Acute care hospitals, general hospitals, and long-term care facilities	Front-line nursing managers	95	MSI; resonant leadership; empowerment; burnout	Workload and difficulty ensuring quality care were associated with burnout and cynicism.
Iguchi (2016) [42]	Japan	Not specified (prior to 2013)	Structural equation modeling	Public health centers	Public health nurses	1766	NBJSQ; UWES; MBI; SF-8	Job demands were associated with burnout and intention to leave; resources reduced burnout.
Jourdain and Chênevert (2010) [43]	Canada	2005	Cross-sectional	Public hospitals	Unionized registered nurses	1636	Caplan; MBI-HSS; Meyer’s commitment; Beck’s health; TIS	Quantitative overload was the main determinant of emotional exhaustion.
Leep-Lazar et al. (2026) [44]	United States	August–November 2024	Cross-sectional	Hospitals	Nurses early in their careers (<5 years)	772	CBI; K10; WHO-5; Blau Rotation Scale	Psychological and emotional demands predicted burnout and intention to leave.
Maniscalco et al. (2025) [45]	Belgium, the Netherlands, and Italy	May–September 2022	Multicenter cross-sectional study (METEOR)	Academic and non-academic hospitals	Physicians	375	MBI; demands; resources; satisfaction; TIS	Workload and lack of equipment were associated with burnout and intention to leave.
Petersen et al. (2023) [7]	Germany	2018 BIBB/BAuA Survey	Secondary analysis of a representative survey	Hospitals, nursing homes, and home care	Nurses	585	Single item on burnout; BIBB/BAuA psychosocial factors	Emotional exhaustion was associated with more days of sick leave per year.
Peterson et al. (2008) [46]	Sweden	Unspecified (prior to 2008)	Cross-sectional	County Council Health Services	Healthcare personnel: physicians, nurses, midwives, and other roles	3719	OLBI; JD-R scales of demands and resources	Burnout was associated with higher rates of absenteeism, presenteeism, and overtime.
Qiao et al. (2026) [34]	China	12-month implementation in 2026	Quasi-experimental with a control group	Regional public hospital	Physicians, nurses, technicians, and administrative staff	600	SPS-6; WHO-5; MBI; NASA-TLX	Workload increased presenteeism both directly and indirectly through well-being and burnout.
Scanlan and Still (2019) [47]	Sydney, Australia	8 months of data; baseline in 2011	Cross-sectional	Mental health services	Mental health staff: physicians, nurses, and other roles	277	OLBI; job satisfaction; TIS; demand and resource scales	Emotional demands predicted burnout, turnover intention, and lower job satisfaction.
Stordeur et al. (2007) [38]	Belgium	2002–2003	Comparison between hospitals with low and high turnover	Twelve Belgian hospitals	Registered nurses	1175	NEXT questionnaire; MBI; WAI; satisfaction; TIS	Hospitals with low turnover showed lower levels of burnout, overload, and emotional demands.
Tan et al. (2024) [10]	Singapore	Not specified (accepted in 2024)	Descriptive-correlational cross-sectional study	Tertiary hospital; hematology	Hematology nurses	60	COPSOQ III; ProQOL5; TIS-6	Cognitive and emotional demands predict compassion fatigue, burnout, and intention to rotate.
Van der Heijden et al. (2019) [16]	Netherlands	Longitudinal with a 1-year lag	Longitudinal	Hospitals, nursing homes, and home care	Registered nurses	1187	De Jonge’s job demands scales; COPSOQ; social support; intention to leave	Job demand increased burnout, which mediated the intention to leave the profession the following year.
Wong et al. (2024) [48]	China	October–December 2022	Cross-sectional	Thirteen public hospitals	Registered nurses	502	Scales of role overload, work stress, work-family conflict, NSS, MBI, salary satisfaction, and turnover intention	Overload, stress, and conflicts were associated with turnover intention mediated by burnout.
Odom-Forren et al. (2025) [49]	United States	January 10–March 11, 2021	Descriptive, exploratory, and cross-sectional	Perioperative and hospital procedure areas	Registered nurses	1436	NSS; ProQOL; retention intention questions; COVID-19 experience	Workload was associated with lower retention intent during the pandemic.
Xian et al. (2020) [50]	China	March–June 2017	Cross-sectional survey	Tertiary hospitals; emergency departments, operating rooms, and ICUs	Male nurses	366	MBI-GS; Chinese Scale of Work Demands and Resources for Nurses	Workload, stress, shifts, and working hours were associated with higher levels of burnout.
Chênevert et al. (2021) [51]	Canada	Not specified	Cross-sectional survey	Hospital settings and medical specialty associations	Physicians	407	Scales measuring work-related stress, burnout, work-family conflict, organizational support, meaning, recognition, MBI-HSS, and work-related outcomes	Emotional exhaustion and depersonalization mediated the intention to leave the medical profession.
Cheyroux et al. (2025) [36]	France	Not specified	Person-centered longitudinal study	Healthcare settings in France	Nurses	622	Questionnaires on job passion, burnout, support, perceived injustice, workplace harassment, GHQ, 3D-WFI, presenteeism, intention to leave, and HPQ	Burnout and perceived injustice were associated with higher turnover, presenteeism, and absenteeism.
Rickard et al. (2012) [35]	Australia	2008 and 2010	Quasi-experimental pre-post evaluation	Public hospitals	Registered nurses and midwives	484	GHQ-12; MBI; UWES-9; job satisfaction; NSS; JCQ; COPSOQ; turnover indicators	The intervention reduced psychological distress and burnout and stabilized turnover.
Barbon et al. (2026) [52]	Italy	Not specified	Cross-sectional survey	Hospitals	Healthcare professionals: nurses, physicians, healthcare assistants, technicians, and administrative staff	802	Adapted NAQ-R; Stress Indicator Tool; COPSOQ; Kelloway Turnover Intention Scale	Workload, time pressures, and interpersonal conflict predicted burnout and turnover intention.
Thapa et al. (2022) [33]	Sweden	2016 and 2018	Prospective longitudinal	Public and private healthcare sectors	Nursing professionals: registered nurses, specialists, midwives, and nursing assistants	1114	Indices of work demands and resources; self-reported health; SMBQ; recorded and self-reported absenteeism	Psychological and emotional demands predicted poorer health, burnout, and absenteeism.
Rotenstein et al. (2023) [22]	United States	April–December 2020	Cross-sectional survey	206 healthcare organizations; inpatient and outpatient care	Physicians, nurses, other clinical staff, and non-clinical staff	43,026	Unique items on COVID-19 overload, Mini-Z burnout, and intention to leave	Overload predicted burnout and intention to leave across all healthcare roles.
Luther et al. (2017) [53]	United States	January 2014–January 2015	Cross-sectional analysis of baseline data from a randomized controlled trial	Community mental health centers	Mental health clinicians	182	Self-reported overtime; MBI-HSS; job satisfaction; intention to leave; work–life conflict; perceived quality of care	Overtime was associated with burnout, work–life conflict, and lower job satisfaction.
Van Bogaert et al. (2017) [54]	Belgium	September 2014–June 2015	Mixed methods	Acute care teaching hospitals	Ward nurses	751	NWI-R; MBI-HSS; UWES; Intensity of Labor Scale; social capital scale; work outcomes reported by nurses	Workload was associated with burnout, lower vigor, and poorer work outcomes.
Chen et al. (2019) [55]	Taiwan	2013–2014	Cross-sectional survey	Medical and surgical departments in 24 secondary and tertiary hospitals	Registered nurses	1409	Patient-to-nurse ratio (ADPNR); C-CBI; job satisfaction item; intention-to-quit scale	A higher patient-to-nurse ratio predicted burnout, dissatisfaction, and turnover intention.
Peter et al. (2024) [56]	Switzerland	September 2017–September 2020	Repeated cross-sectional design	Acute care hospitals, rehabilitation facilities, psychiatric hospitals, nursing homes, and home care	Healthcare professionals in various clinical, technical, therapeutic, administrative, and social roles	19,340	STRAIN questionnaire; COPSOQ; EWCS; EQ-5D-5L; NEXT/COPSOQ scales	Work–life imbalance was the most consistent predictor of stress, burnout, and intention to leave.
Adriaenssens et al. (2017) [9]	Belgium	2015	Cross-sectional	Eleven secondary acute care hospitals	Front-line nursing managers	318	LQWQ; QEAW; MBI-HSS; BSI; UWES; satisfaction and intention to leave	Time pressure and work pressure predicted burnout, dissatisfaction, and psychosomatic distress.
Moloney et al. (2018) [3]	New Zealand	2014–2015	Cross-sectional	Various healthcare settings	Registered nurses	2876	BMS-10; UWES-9; scales measuring workload, emotional demands, work–life conflict, work and personal resources; intention to leave	Work overload and work–life conflict were the strongest predictors of burnout and risk of turnover.
Vandenbroeck et al. (2017) [2]	Belgium	June–July 2012	Multicenter cross-sectional study	37 general and psychiatric hospitals	Doctors and Nurses	5700	UBOS; SIMPH; NWI-R; WOCCQ; SF-20; SF-36; indicators of turnover intention, absenteeism, and presenteeism	Emotional exhaustion was associated with job demands, intention to leave, absenteeism, and presenteeism.

### 3.2. Narrative Synthesis of the Constructs, Instruments, and Key Associations Identified in the Review

#### 3.2.1. Conceptual Aspects

Consistent with the Job Demands–Resources model (JD-R) [5], job demands encompass physical, psychological, social, or organizational aspects of work that require sustained effort and entail physiological or psychological costs [2,3,9,35,50]. Work overload is a quantitative job demand that refers to the perception of having more work than can be completed within a given time frame [7,8]. Mental workload refers to the cognitive and information-processing effort required by tasks involving concentration, memory, or the simultaneous management of information [9,10].

Although mental workload was included in several studies, it received less empirical attention and produced more heterogeneous results than work overload [10,47]. Some findings suggest that cognitive demands may function as challenge demands under certain conditions, whereas excessive workload more consistently operates as a hindrance demand [47].

Burnout, the predominant construct in the included studies, is conceptualized as a chronic syndrome resulting from prolonged occupational stressors that have not been successfully managed [9,50,55]. Three main approaches were used to assess burnout: the three-dimensional model measured by the Maslach Burnout Inventory, which includes emotional exhaustion, depersonalization, and reduced personal accomplishment [2,9,51]; the Oldenburg Burnout Inventory, which assesses exhaustion and detachment [46,47]; and emotional exhaustion as a core dimension that appears particularly sensitive to quantitative job demands [7,43].

Work-related fatigue refers to extreme exhaustion resulting from the workday, whereas burnout represents a deeper and more persistent response to prolonged occupational stress [35,46]. The Three-Dimensional Work Fatigue Inventory (3D-WFI) enables the assessment of physical, mental, and emotional fatigue at the end of the workday [12,36]. Within the JD-R model, severe fatigue forms part of the health-impairment process triggered by excessive job demands and may precede burnout [8,35].

Turnover intention is the most widely studied form of job departure. It refers to the conscious and deliberate likelihood of leaving one’s job soon [8,52] and is considered a proximal antecedent of actual turnover [38,43,52]. It may arise from resource erosion processes mediated by emotional exhaustion and job dissatisfaction [10,16,38,55]. As noted earlier, it is important to distinguish between intention to leave a unit or hospital and intention to leave the profession, the latter being particularly consequential for healthcare systems [2,3,57].

Actual turnover has been less frequently examined because it is an observed outcome that generally requires longitudinal follow-up or administrative records. It represents the most explicit manifestation of job departure. One Belgian study reported that 19.7% of emergency department nurses left their positions within 18 months [32,38]. Beyond recruitment and training costs, turnover may compromise continuity and quality of care [10,48,52].

Absenteeism is closely associated with chronic burnout. Professionals with high levels of emotional exhaustion are significantly more likely to report more than 10 sick days per year [7,33]. Psychological and physical demands are among the strongest predictors of absenteeism [33,46]. Presenteeism, by contrast, refers to attending work despite health problems that would justify absence [34,46]. It is highly prevalent in the healthcare sector and has been associated with an increased risk of medical errors, patient safety incidents, and subsequent burnout [2,34].

#### 3.2.2. Measurement Instruments

For burnout, the Maslach Burnout Inventory (MBI) is the most widely used; its MBI-HSS version measures emotional exhaustion, depersonalization, and personal fulfillment [9,43]. Other measures include the Oldenburg Burnout Inventory (OLBI: exhaustion and detachment) [7,47], the Copenhagen Burnout Inventory (CBI: personal, work-related, and patient-related exhaustion) [16,55], and the Shirom–Melamed Burnout Questionnaire (SMBQ), which focuses on physical and cognitive exhaustion [46].

Workload and psychological demands were measured using both general and nursing-specific instruments: the COPSOQ for quantitative, cognitive, and emotional demands [7,10,33]; the Nursing Stress Scale (NSS) for stressors in hospital practice [48,49]; the LQWQ-N for job characteristics and well-being in nursing [32,38]; and the NASA-TLX for perceived workload in high-criticality tasks [52].

Intention to leave a job, unit, or organization was primarily measured using brief scales of three to six items, including the Turnover Intention Scale (TIS-6) [10]. Other studies used the Blau Scale or items adapted from the European NEXT (Nurses’ Early Exit Study) questionnaire [2,43].

#### 3.2.3. Narrative Synthesis of Associations

Workload/Work Overload. Quantitative work overload was assessed in 18 studies (15 cross-sectional and 3 longitudinal) using quantitative demand subscales from the Copenhagen Psychosocial Questionnaire (COPSOQ) [7,10], the Job Content Questionnaire (JCQ) [43], the Social Inventory to Monitor Psychosocial Hazards (SIMPH) [2,32], the Nursing Stress Scale (NSS) [48], and ad hoc measures of perceived workload [8,51,52].

According to the predefined decision rule, the evidence was classified as consistent/conclusive (100% of studies). Work overload showed direct, positive, and statistically significant associations with emotional exhaustion/burnout (r ranging from 0.31 to 0.72, *p* < 0.001; β ranging from 0.25 to 0.60, *p* < 0.001) and turnover intention (r ranging from 0.06 to 0.37, *p* < 0.05). Longitudinal studies further indicated that baseline work overload predicted significant increases in exhaustion at 12- and 18-month follow-up assessments [16,32].

The main methodological limitations included the potential for common method variance arising from the concurrent assessment of workload and exhaustion using self-administered questionnaires, as well as the limited use of objective workload indicators, such as patient-to-nurse ratios or patient acuity measures.

Mental Workload/Cognitive Demands. Mental workload and cognitive demands were assessed in seven studies (six cross-sectional and one longitudinal) using COPSOQ subscales [10,39,40,47,56] and the NASA Task Load Index (NASA-TLX) [34,52].

Evidence regarding their direct associations with exhaustion and turnover intention was classified as mixed/inconsistent. While studies conducted in highly demanding work environments reported that mental workload was associated with greater psychological distress when combined with low job control [10,40], Scanlan and Still (47) found no statistically significant correlation between cognitive demands and exhaustion (r = −0.02), but reported a weak positive association with job satisfaction (r = 0.12, *p* < 0.05).

These findings suggest that mental workload may function as a challenge stressor rather than a hindrance stressor under certain conditions within the JD-R framework. The main limitation was substantial heterogeneity in the operational definitions and measurement of this construct.

Work-Related Fatigue. Work-related fatigue was examined in five studies (three cross-sectional and two longitudinal) using the Three-Dimensional Work Fatigue Inventory (3D-WFI) [36], the Shirom–Melamed Burnout Measure/Questionnaire (SMBM/SMBQ) [8,33], and the exhaustion subscale of the Oldenburg Burnout Inventory (OLBI) [46,47].

According to the predefined decision rule, the evidence was classified as consistent/conclusive (100% of studies). Job demands were positively associated with physical, mental, and emotional fatigue (β ranging from 0.21 to 0.44, *p* < 0.01; r ranging from 0.34 to 0.56, *p* < 0.001).

The main methodological limitations included the predominant reliance on self-reported fatigue measures and the limited assessment of within-day fluctuations or the use of ecological momentary assessment (EMA) during work shifts.

Absenteeism/Sickness Absence. Absenteeism was assessed in seven studies (four cross-sectional and three longitudinal), using both administrative records and self-reported measures.

Administrative or objective records (k = 3). Sickness absence was examined using hospital or institutional records [34,37] or national social insurance registers [33]. The evidence was classified as consistent, indicating that quantitative job demands and exhaustion predicted higher levels of recorded sickness absence. Thapa et al. [33] reported an odds ratio of 1.89 (95% CI [1.13, 3.13]).

Self-reported measures (k = 4). Absenteeism was assessed using retrospective 12-month self-report questionnaires or items from the World Health Organization Health and Work Performance Questionnaire (WHO-HPQ) [2,7,36,46]. The evidence was classified as consistent, with healthcare professionals experiencing high burnout reporting greater frequencies of sickness absence exceeding 10 or 25 days per year. Peterson et al. [46] reported a higher proportion of sickness absence among workers with burnout than among those without burnout (13.8% vs. 5.9%).

The main limitations of self-reported measures included potential recall bias, whereas administrative records did not necessarily allow absences attributable to occupational conditions to be distinguished from those arising from non-occupational health problems.

Presenteeism. Presenteeism was examined in five studies (three cross-sectional, one longitudinal, and one quasi-experimental) using the Stanford Presenteeism Scale (SPS-6) [34], annual self-reported measures [46], and ad hoc scales [2,36].

According to the predefined decision rule, the evidence was classified as consistent/conclusive (100% of studies). Work overload and exhaustion were positively associated with presenteeism (β ranging from 0.39 to 0.42, *p* < 0.001) [34]. Peterson et al. [46] reported a higher prevalence of presenteeism among workers experiencing burnout than among those without burnout (59.2% vs. 26.6%).

Qiao et al. [34] additionally assessed presenteeism using objective clinical performance records, reporting an association between reductions in presenteeism and lower medical error rates (r = −0.28, *p* < 0.01).

Actual Turnover. Actual turnover was examined in three longitudinal or cohort studies [32,38,58] using institutional personnel records over follow-up periods of 12 or 18 months.

Adriaenssens et al. [32] reported an actual turnover rate of 19.7% at 18 months, whereas Stordeur et al. [58] reported annual turnover rates ranging from 0.6% to 13.1%. The evidence was classified as consistent, indicating that higher baseline job demands and emotional exhaustion were associated with an increased likelihood of recorded actual turnover.

The main methodological limitations included the relatively low base rate of actual turnover events and attrition during longitudinal follow-up.

Table 2 summarizes the narrative evidence across the examined constructs, including study designs, measurement approaches, directions and magnitudes of associations, and principal methodological limitations.

### 3.3. Meta-Analysis

For quantitative synthesis, studies were selected according to the following criteria:

1. Explicit assessment of work overload or quantitative demands, as this is the factor for which the most comparable estimates are available across the set of included studies.
2. Assessment of overall burnout or, alternatively, of emotional exhaustion as the central and affective component of the construct. We have considered measures of general exhaustion and its primary dimension, emotional exhaustion, since the latter represents the central component of stress and the immediate energetic response to job demands, according to the “Job Demands and Resources” (JD-R) framework.
3. Assessment of turnover intention or intention to leave the organization clearly distinguished from resignation, absenteeism, retention intention, or other organizational outcomes, since this outcome presented sufficient conceptual and statistical homogeneity for quantitative synthesis.
4. Explicit reporting of zero-order correlation coefficients (Pearson’s r) accompanied by sample size (N). Nonparametric correlation coefficients (Spearman’s r) or other measures of association (beta coefficients, odds ratios, or others) were excluded.
5. Ability to verify and identify the independence of the reported samples.

A complete overview of all full-text evaluated studies—including the extracted zero-order correlation matrices (r) and sample sizes for included primary studies, as well as detailed study-by-study exclusion justifications for non-eligible reports—is provided in Supplementary Table S1.

Five of the 34 studies provided six independent samples with comparable data for estimating three associations between work overload, burnout, and turnover intention. Correlation coefficients were transformed using Fisher’s z-transformation, and random-effects models were estimated using restricted maximum likelihood (REML) to model between-study variance (Table 3).

To facilitate graphical interpretation across all bivariate meta-analyses, primary study effect estimates in the forest plots (Figures 2, 4, and 6) are represented by solid black squares, with square areas proportional to study statistical weights under the random-effects model. Horizontal lines denote 95% confidence intervals (CIs). The overall pooled effect size is indicated by the center of the open diamond, with its lateral tips representing the 95% CI. The solid horizontal bar beneath the diamond depicts the 95% Prediction Interval (PI), illustrating the expected dispersion of true effects in future settings. Publication bias was visually examined using residual funnel plots (Figures 3, 5, and 7), where study precision (standard error) is plotted against centered residual effect sizes within pseudo-95% confidence boundaries.

#### 3.3.1. Association Between Work Overload and Burnout or Emotional Exhaustion

The mean effect size estimated using the random-effects model and back-transformed to Pearson correlation was r = 0.58 (95% CI: 0.55 to 0.61; 95% PI: 0.55 to 0.61), corresponding to z = 0.67 on Fisher’s scale (SE = 0.02; z = 37.03, *p* < 0.001) (see Figure 2).

The heterogeneity analysis showed no statistically significant variability among the true effects of the studies (Q(5) = 4.60, *p* = 0.47; τ^2^ = 0.00; I^2^ = 0.02%).

The sensitivity analysis demonstrated stability in the findings. The identification of outliers and influential cases (studentized residuals < 2.64) revealed the absence of outlier studies in the model.

The assessment of funnel plot visual symmetry and formal statistical tests revealed no statistically significant evidence of funnel plot asymmetry (see Figure 3). Specifically, neither the Begg and Mazumdar rank correlation test (τ = −0.33, *p* = 0.47) nor the Egger regression test (t = −1.07, *p* = 0.29) showed statistically significant asymmetry. Rosenthal’s Fail-Safe N was 2872 (*p* < 0.001), indicating a large tolerance to potential unpublished null findings. However, these results must be interpreted with caution: given the small number of included studies (k = 6), the statistical power of funnel plots, regression tests, and Fail-Safe N metrics to detect publication bias is limited, and the lack of statistical evidence of asymmetry does not definitively rule out the possibility of publication bias.

#### 3.3.2. Association Between Burnout and Turnover Intention

The weighted average effect size derived from the random-effects model, back-transformed to Pearson correlation, was r = 0.41 (95% CI: 0.34 to 0.48; 95% PI: 0.24 to 0.56), corresponding to z = 0.44 on Fisher’s scale (SE = 0.03; z = 12.85, *p* < 0.001) (see Figure 4).

Cochran’s Q test detected the presence of statistically significant heterogeneity among the studies (Q(5) = 17.78, *p* = 0.003; τ^2^ = 0.00; I^2^ = 70.21%).

The sensitivity analysis showed that the studentized residuals remained within the limits established by the Bonferroni correction (<2.64), revealed the absence of outliers or cases with a disproportionate impact on the point estimate. Despite the presence of heterogeneity among the studies, the direction and significance of the average effect remained stable in the sensitivity analyses, supporting the validity of the model.

The assessment of funnel plot visual symmetry and formal statistical tests revealed no statistically significant evidence of funnel plot asymmetry (see Figure 5). Specifically, neither the Begg and Mazumdar rank correlation test (tau = −0.07, *p* = 1.00) nor the Egger regression test (t = −0.080, *p* = 0.43) showed statistically significant asymmetry. Rosenthal’s Fail-Safe N index was 1262 studies (*p* < 0.001), indicating a high tolerance to potential unpublished null findings. However, these results must be interpreted with caution.

#### 3.3.3. Association Between Work Overload and Turnover Intention

The point estimate of the mean effect under the random-effects model, back-transformed to Pearson correlation, was r = 0.28 (95% CI: 0.22 to 0.35; 95% PI: 0.15 to 0.41), corresponding to z = 0.29 on Fisher’s scale (SE = 0.03; z = 10.66, *p* < 0.001) (see Figure 6).

The heterogeneity test did not reach conventional statistical significance, although the I^2^ statistic suggested moderate between-study variability (Q(5) = 10.72, *p* = 0.06; τ^2^ = 0.00; I^2^ = 53.61%).

To assess the model’s sensitivity, studentized residuals and influence metrics were analyzed. No study exceeded the Bonferroni-corrected critical threshold (<2.64). No outliers were identified using the prespecified diagnostic threshold. Although the model exhibited moderate heterogeneity, the diagnostics confirmed the absence of cases that influenced the point estimate of the overall effect.

The assessment of funnel plot visual symmetry and formal statistical tests revealed no statistically significant evidence of funnel plot asymmetry (see Figure 7). Specifically, neither the Begg and Mazumdar rank correlation test (tau = −0.07, *p* = 1.00) nor the Egger regression test (t = −0.065, *p* = 0.52) showed statistically significant asymmetry. Rosenthal’s Fail-Safe N index was 548 studies (*p* < 0.001), indicating tolerance to potential unpublished null findings. However, these results must be interpreted with caution.

Taken together, these findings indicate that the associations involving work overload were comparatively more consistent across studies than the burnout–turnover intention association. Whereas work overload showed highly homogeneous associations with burnout and a comparatively more consistent positive association with turnover intention, the burnout–turnover intention association displayed substantial heterogeneity, suggesting greater sensitivity to organizational, geographic, sectoral, or measurement-related contexts.

#### 3.3.4. Assessment of the Certainty of Evidence for the Meta-Analytic Associations

The certainty of evidence was assessed using the GRADE approach for each of the three meta-analytic associations. Because the meta-analytic evidence was derived from observational studies, the initial certainty was rated as low under the conventional GRADE approach. The JBI assessments were used to inform the subsequent evaluation of risk of bias.

Certainty was subsequently evaluated across the GRADE domains of risk of bias, inconsistency, indirectness, imprecision, and publication bias, and potential upgrading criteria were also considered.

No additional downgrade for risk of bias was applied. Although the evidence was predominantly cross-sectional and the meta-analyses were based on unadjusted zero-order correlations, concerns regarding residual confounding and the absence of randomization were considered inherent limitations of the observational evidence and were therefore reflected in the initial low-certainty rating. The JBI assessments indicated that four of the five publications contributing to the meta-analyses identified confounding factors and reported strategies to address them, whereas one publication, contributing two independent samples, did not. However, the pooled estimates were based on unadjusted correlations, and therefore residual confounding could not be excluded. Differences in measurement approaches across studies were also considered but were not judged sufficient to warrant an additional downgrade.

The work overload–burnout association was rated as low-certainty evidence (⊕⊕◯◯), with no additional downgrading for inconsistency, indirectness, or imprecision. This association showed the lowest between-study heterogeneity among the three meta-analyses, indicating comparatively consistent findings. Sensitivity analyses did not identify individual estimates with excessive statistical influence, supporting the stability of the pooled association. The burnout–turnover intention association was downgraded one additional level for serious inconsistency because of substantial between-study heterogeneity (I^2^ = 70.21%; Q(5) = 17.78), resulting in very-low-certainty evidence (⊕◯◯◯). Although sensitivity diagnostics did not identify estimates exceeding the Bonferroni-adjusted outlier threshold, this finding did not eliminate concerns regarding between-study inconsistency. The work overload–turnover intention association was rated as low-certainty evidence (⊕⊕◯◯). Although moderate heterogeneity was observed (I^2^ = 53.61%; Q(5) = 10.72), effects were consistently positive and the prediction interval remained above the null, and inconsistency was therefore not judged serious. Furthermore, sensitivity analyses did not identify individual estimates exerting excessive influence on the pooled result.

Assessment of publication bias was considered inconclusive for all three associations because only six independent samples from five publications were available. Consequently, funnel plots and statistical tests of asymmetry had limited power, and non-significant Begg and Egger tests were not interpreted as evidence of absence of publication bias. Although no statistically significant evidence of funnel plot asymmetry was detected, publication bias could not be ruled out. No upgrading of certainty was applied because the available evidence did not provide sufficiently robust support for a large-effect, dose–response, or residual-confounding criterion (Table 4).

### 3.4. Structural Meta-Analytic Mediation Model (TSSEM)

To explore the proposed indirect association, the Two-Stage Structural Equation Modeling (TSSEM) approach [59] was used, employing Weighted Least Squares (WLS) estimation via the metaSEM package in R. Methodologically, the synthetic correlation matrix used in the Two-Stage Structural Equation Modeling (TSSEM) was constructed from the same six independent primary samples (comprising five publications: [8,43,47,51,52]). Because each of these six samples explicitly reported complete 3 × 3 zero-order correlation matrices among work overload, burnout, and turnover intention, no incomplete correlation matrices or missing value imputations were present. Furthermore, using the harmonic mean of the sample sizes (N = 1150) as the Stage 2 sample size represents the standard, conservative convention in TSSEM methodology [59]. The harmonic mean penalizes sample size dispersion across primary studies, preventing larger studies from artificially inflating total statistical power or underestimating parameter standard errors.

The estimated structural parameters revealed that work overload was strongly associated with burnout (β = 0.58, SE = 0.019, z = 29.89, *p* < 0.001, 95% CI: [0.544, 0.621]), which in turn was positively associated with turnover intention (β = 0.37, SE = 0.031, z = 11.88, *p* < 0.001, 95% CI: [0.312, 0.435]). When burnout was included in the model, the direct association between work overload and turnover intention was substantially attenuated from a total association of β = 0.28 (SE = 0.038, z = 7.40, *p* < 0.001, 95% CI: [0.208, 0.358]) to a residual direct association of β = 0.07 (SE = 0.033, z = 1.99, *p* = 0.047, 95% CI: [0.001, 0.130]).

Parameter estimation via matrix algebra (mxAlgebra) revealed a statistically significant indirect association between work overload and turnover intention through burnout (β_indirect = 0.22, SE = 0.019, z = 11.21, *p* < 0.001, 95% CI: [0.180, 0.256]), which accounted for 76.8% of the total modeled association (see Table 5). The structural association pattern is depicted in the path diagram in Figure 8. Single-headed arrows denote directional specifications of regression paths within the model rather than established temporal or causal mechanisms.

Given the limited number of independent samples (k = 6), these TSSEM findings should be considered exploratory, as the small evidence base restricts the stability and generalizability of the estimated structural parameters.

## 4. Discussion

This systematic review and meta-analysis synthesized evidence published between 2007 and 2026 on the relationship between work overload, burnout, and withdrawal behaviors among healthcare workers. The results show a pattern in which work overload is associated with higher levels of burnout, and burnout is strongly related to turnover intention. The direct association between work overload and turnover intention is attenuated when burnout is included in the model. This pattern is consistent with previous research showing associations of work overload and mental workload with adverse occupational outcomes among healthcare workers, particularly in contexts of high care demands, time pressure, insufficient staffing, and high emotional demands [2,14,16,60,61].

Therefore, at the pooled level, the findings support an associative pattern linking work overload, burnout, and turnover intention, without establishing the temporal or causal ordering of these variables.

The results are consistent with the Job Demands and Resources model, as job demands were associated with burnout and organizational resources with more favorable outcomes. Time pressure, overload, lack of staffing, work-family conflict, and emotional demands have been associated with higher burnout, whereas social support, autonomy, recognition, and organizational resources have been associated with more favorable occupational outcomes [2,8,14,16,51]. Although these findings are consistent with the JD-R framework, the present meta-analysis focused on the associations between work overload, burnout, and turnover intention and did not evaluate the full health-impairment and motivational pathways proposed by the model.

Beyond the pooled associations, individual studies included in the review provide evidence on related occupational demands and withdrawal outcomes that could not be quantitatively synthesized. Mental workload and work-related fatigue were examined less consistently than burnout, while individual studies reported associations involving absenteeism, presenteeism, and turnover-related outcomes [4,7,11,14,18,19,34,46]. These findings broaden the pattern observed in the meta-analysis but should be interpreted at the level of the original study designs and do not establish a common temporal sequence across these outcomes.

Beyond job demands and resources, burnout may also be associated with intraindividual, interpersonal, and group-level factors. Among Greek nurses, workplace mobbing was positively associated with emotional exhaustion, whereas perceived group cohesion was negatively associated with mobbing [62]. Other characteristics, such as hubris, have also been explored, although preliminary findings did not identify a significant association with burnout [63]. These findings highlight the multifactorial nature of burnout and the need for broader multilevel approaches.

A worker’s age, career stage, and professional context can influence vulnerability to burnout and turnover intention. Previous studies have reported associations involving early-career healthcare professionals and specific occupational demands among male nurses or workers in high-complexity units [10,32,39,44,60,61]. These represent potential avenues for future research and were not evaluated here through subgroup analyses or meta-regression.

Consistent with this broader perspective, internal workplace dynamics may also influence withdrawal-related outcomes. Although external violence from patients may play a role, internal conflicts and a lack of support from supervisors and colleagues have been associated with turnover-related outcomes [38,52]. Serial mediation suggests that a deteriorating social environment is associated with lower job satisfaction and greater vulnerability to job demands [38]. Burnout may be relevant to both worker well-being and healthcare quality, given its reported associations with turnover intention and presenteeism [36,49]. Healthcare workforce retention may have important implications for patient safety and organizational sustainability [30,36].

This review identified significant gaps in the literature. Work-related fatigue has received far less attention than burnout, preventing a quantitative assessment of its association with work withdrawal. Furthermore, the heavy concentration of research in Europe and North America limits cross-cultural generalizability, underscoring the critical need for longitudinal studies in underrepresented regions such as Latin America.

The three bivariate meta-analyses revealed differences in between-study heterogeneity. The burnout–turnover intention association showed substantial heterogeneity, potentially reflecting differences in organizational contexts, healthcare settings, or measurement approaches. However, these explanations were not formally tested. In contrast, the work overload–burnout association showed minimal heterogeneity, whereas the work overload–turnover intention association showed moderate heterogeneity.

### 4.1. Limitations of the Present Study

Limitations of the review process itself include search criteria restricted to literature retrieved exclusively from Web of Science (WoS), Scopus, and PubMed, and to studies published in English and Spanish. Additionally, the gray literature (such as unpublished doctoral dissertations or conference proceedings) was not systematically searched, which may introduce potential database, language, or publication selection constraints and may have resulted in relevant evidence being missed. Because only six independent samples were available for each meta-analysis, the statistical tests used to assess publication bias had limited power. Therefore, the absence of statistically significant asymmetry should not be interpreted as evidence of the absence of publication bias, and publication bias could not be reliably ruled out.

Regarding the meta-analyses conducted, only five primary studies, providing six independent samples, reported sufficiently comparable data in terms of constructs, instruments, and measurement systems. Although the cumulative sample sizes allowed precise point estimates, the small number of studies identified and used here limits the statistical power to perform meta-regressions or subgroup analyses that could explain the high heterogeneity found, for example, in the burnout-turnover intention pathway. This limitation is consistent with previous findings that point to marked methodological heterogeneity in research on job demands, burnout, absenteeism, and resignation among healthcare workers [4,20,64,65].

The predominance of cross-sectional designs necessitates interpreting the meta-analyses and the TSSEM as associative and exploratory evidence. Although the pattern is consistent with the work overload–burnout–turnover intention sequence, it does not establish causality or temporality. In addition, the pooled estimates were based on zero-order correlations and therefore do not account for potential confounding. Differences in the operationalization and measurement of work overload, burnout, and turnover intention across the primary studies should also be considered when interpreting the pooled associations. The TSSEM used WLS estimation and the harmonic mean of the sample sizes (N = 1150) and therefore depends on the scale of the available primary studies.

The limited number of independent samples (k = 6) also reduces the precision of heterogeneity estimates, constrains the GRADE assessment of inconsistency and imprecision, and limits the robustness of the exploratory TSSEM. Furthermore, the search strategy may not have identified studies examining only individual pairwise associations, potentially limiting the scope of the meta-analytic findings.

The review was not prospectively registered in PROSPERO, and no formal protocol was published. Therefore, analytical decisions regarding study selection for the meta-analysis and the exploratory TSSEM cannot be compared against a prespecified protocol, which should be considered when interpreting the findings.

Future studies should employ longitudinal designs, temporal mediation analyses, more standardized measures, and objective records of turnover or absenteeism to evaluate the work overload–burnout–turnover intention sequence and its translation into actual turnover or absences. In particular, the substantial heterogeneity observed in the burnout–turnover intention association highlights an opportunity to improve methodological consistency through more standardized measurement instruments, operational definitions, and research procedures. Greater comparability across studies would facilitate more robust meta-analytic syntheses and strengthen the certainty of future evidence. In addition, future research should explore the potential role of leadership in these relationships, particularly whether it may act as a moderator or mediator between work demands and withdrawal behaviors.

### 4.2. Practical Implications for Human Resource Management

Policies focused solely on redistributing workloads may be insufficient without strategies to prevent burnout. Organizational responses could combine staffing and workload management with supervisory and peer support, recognition, autonomy, and work–life balance, which have been associated with more favorable occupational outcomes [2,8,14,16,32,38,51]. Retention may be considered an organizational priority in relation to employee well-being, patient safety, and institutional sustainability [7,11,19,22].

From a practical perspective, healthcare organizations could systematically assess workload, staffing levels relative to service demand, overtime, and time pressure across professional groups and organizational units. Units experiencing sustained overload could be examined jointly by managers and staff before selecting staffing or workflow adjustments. Repeated assessments of burnout using a consistent, validated instrument could be complemented by surveys distinguishing intentions to leave the unit, organization, or profession. Where feasible, anonymous survey trends could be compared with subsequent aggregated records of actual turnover and sickness absence.

Organizational responses should consider locally relevant resources and workplace conditions. Changes in staffing or workflow could be evaluated using pre- and post-implementation measures of employee well-being and recorded workforce outcomes. These recommendations represent organizational actions to be evaluated rather than interventions demonstrated by the present meta-analysis to reduce actual turnover.

## 5. Conclusions

This systematic review and meta-analysis identified significant associations—after pooling the results—between work overload and burnout, between burnout and turnover intention, and between work overload and turnover intention among healthcare workers. From an exploratory perspective, the TSSEM suggests a possible indirect association between work overload and intention to leave via burnout. This finding is consistent with the conclusions of previous studies [2,14,16,60,61].

Regarding the evidence on mental workload, work-related fatigue, absenteeism, presenteeism, and actual turnover, although the studies suggest a relatively similar pattern of relationships, this study examined them only through a narrative synthesis due to methodological and measurement differences among the studies considered.

## Figures and Tables

**Figure 1:**
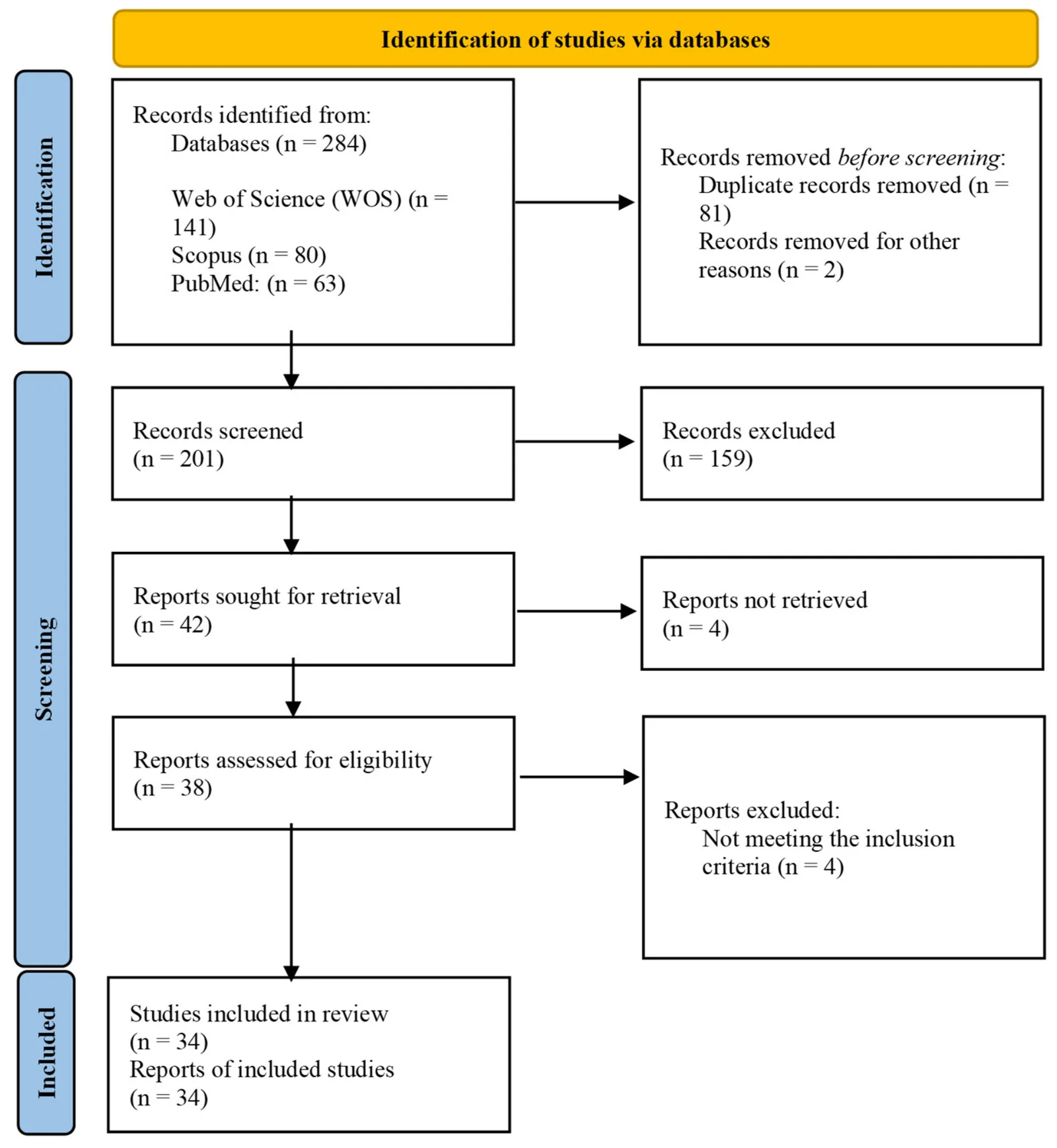
PRISMA 2020 flow diagram of the systematic review process based on the database search. Note: PRISMA 2020 flow diagram depicting the study selection process from initial record identification to final inclusion.

**Figure 2:**
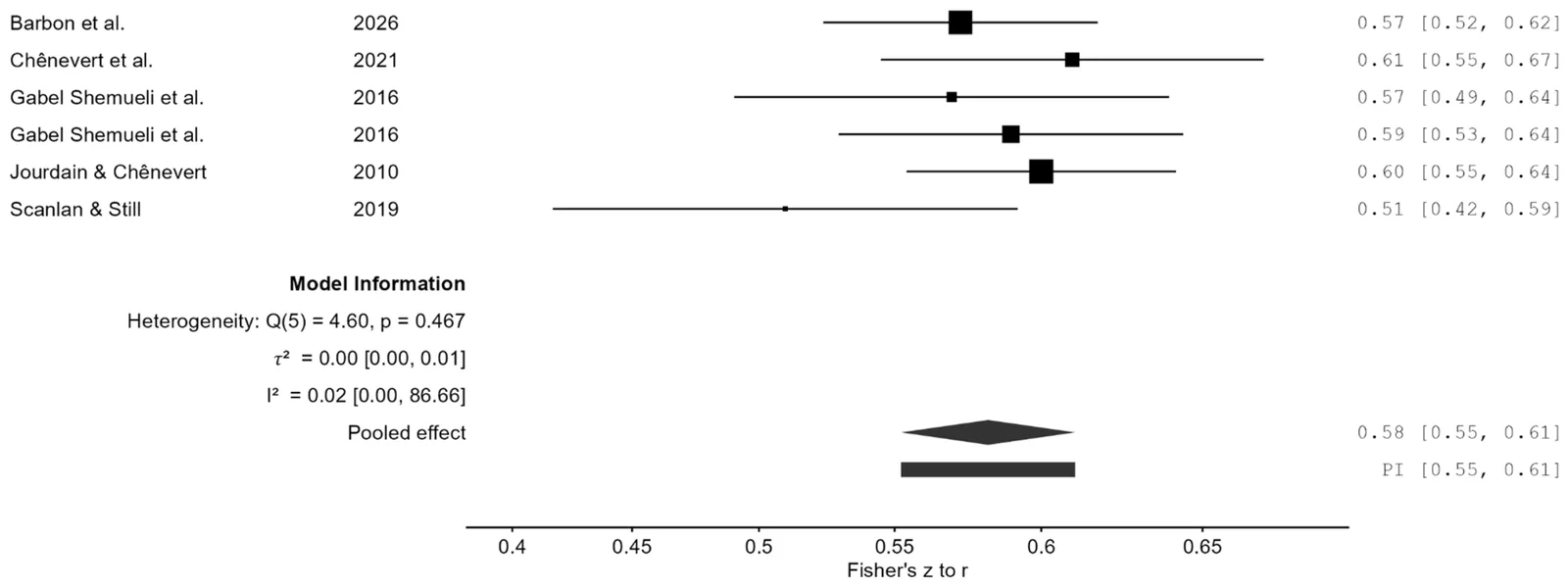
Forest Plot: Meta-analysis of Workload and Burnout. Note: Forest plot of the random-effects meta-analysis for Workload and Burnout (k = 6). Black squares denote individual study estimates (size indicates sample weight); horizontal lines represent 95% CIs; open diamond indicates pooled effect size; bottom horizontal bar represents the 95% Prediction Interval (PI).

**Figure 3:**
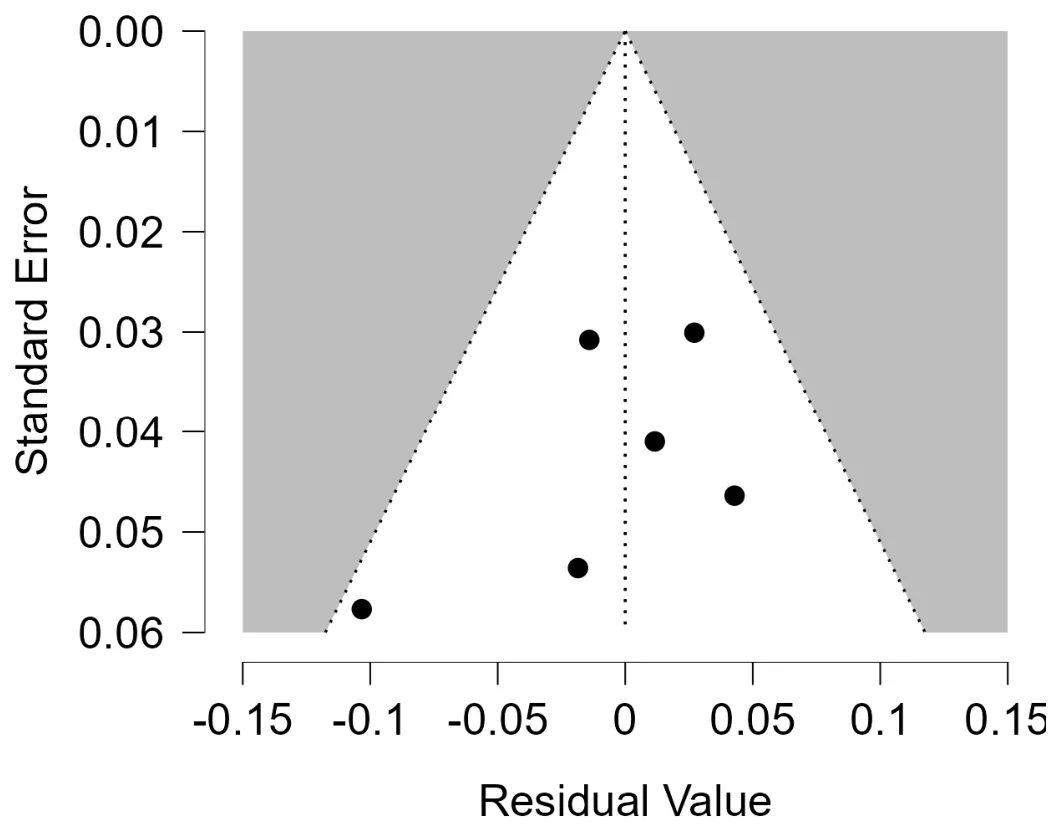
Residual Funnel Plot: Meta-Analysis of Work Overload and Burnout. Note. Residual funnel plot for Work Overload and Burnout. Vertical axis shows standard error (precision); horizontal axis displays residual effect sizes centered at zero. Dashed lines outline pseudo-95% confidence boundaries.

**Figure 4:**
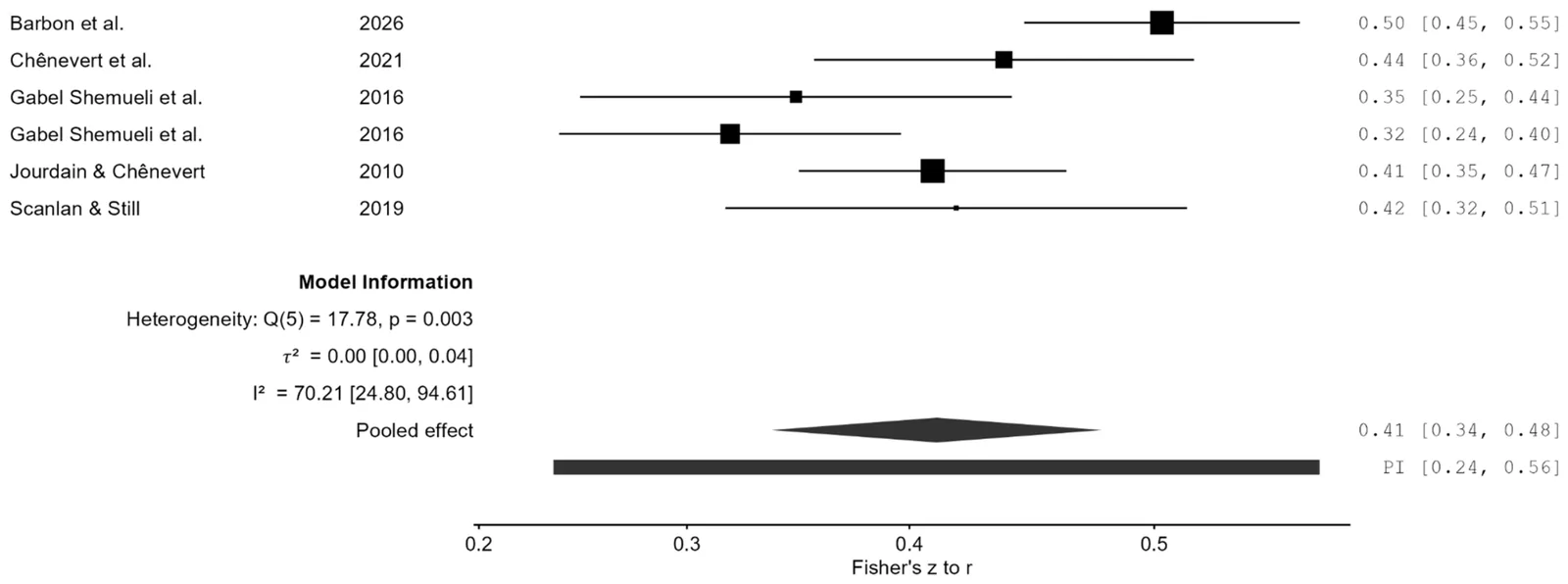
Forest Plot: Meta-analysis of Burnout and Turnover Intention. Note: Forest plot of the random-effects meta-analysis for Burnout and Turnover Intention (k = 6). Black squares denote individual study estimates (size indicates sample weight); horizontal lines represent 95% CIs; open diamond indicates pooled effect size; bottom horizontal bar represents the 95% Prediction Interval (PI).

**Figure 5:**
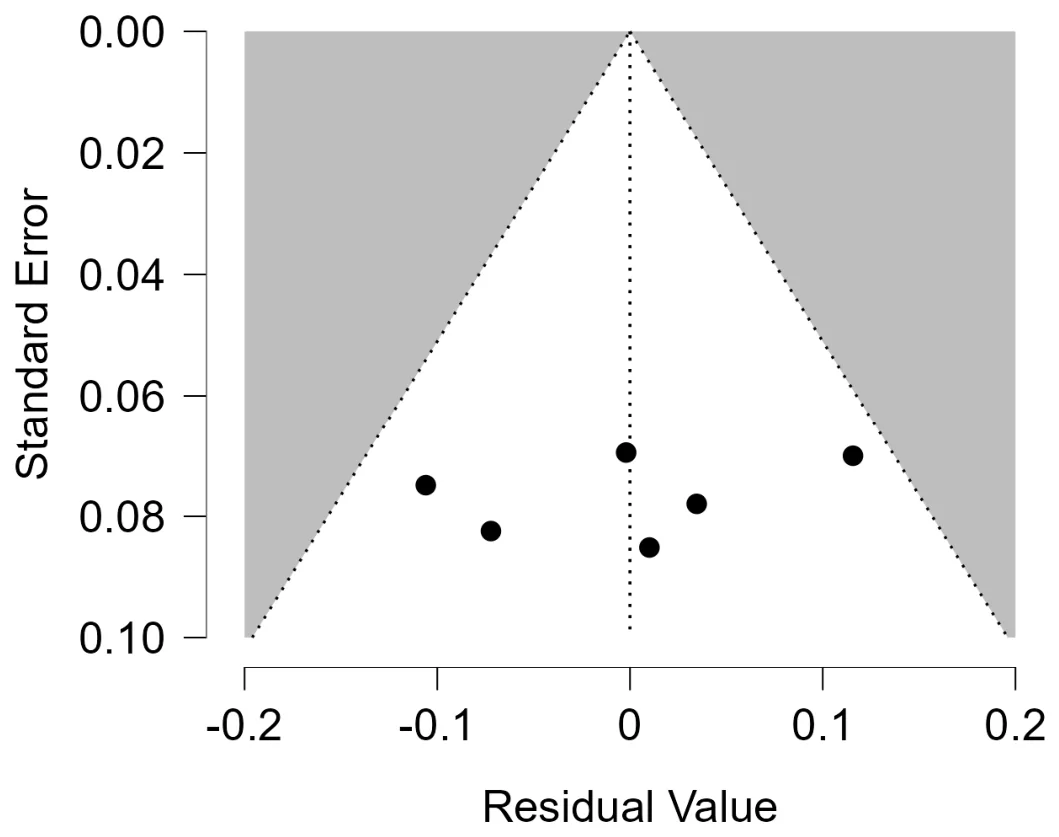
Residual Funnel Plot: Meta-analysis of Burnout and Turnover Intention. Note. Residual funnel plot for Burnout and Turnover Intention. Vertical axis shows standard error (precision); horizontal axis displays residual effect sizes centered at zero. Dashed lines outline pseudo-95% confidence boundaries.

**Figure 6:**
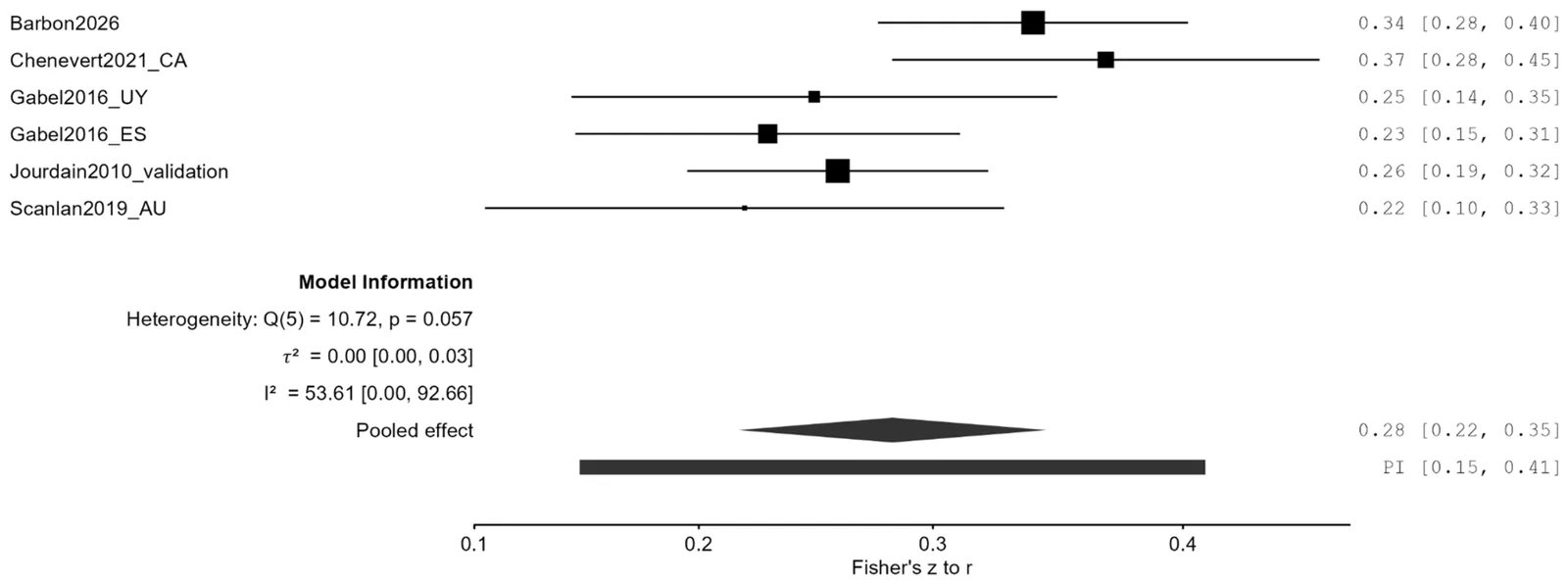
Forest Plot: Meta-analysis of Work Overload and Turnover Intention. Note: Forest plot of the random-effects meta-analysis for Work Overload and Turnover Intention (k = 6). Black squares denote individual study estimates (size indicates sample weight); horizontal lines represent 95% CIs; open diamond indicates pooled effect size; bottom horizontal bar represents the 95% Prediction Interval (PI).

**Figure 7:**
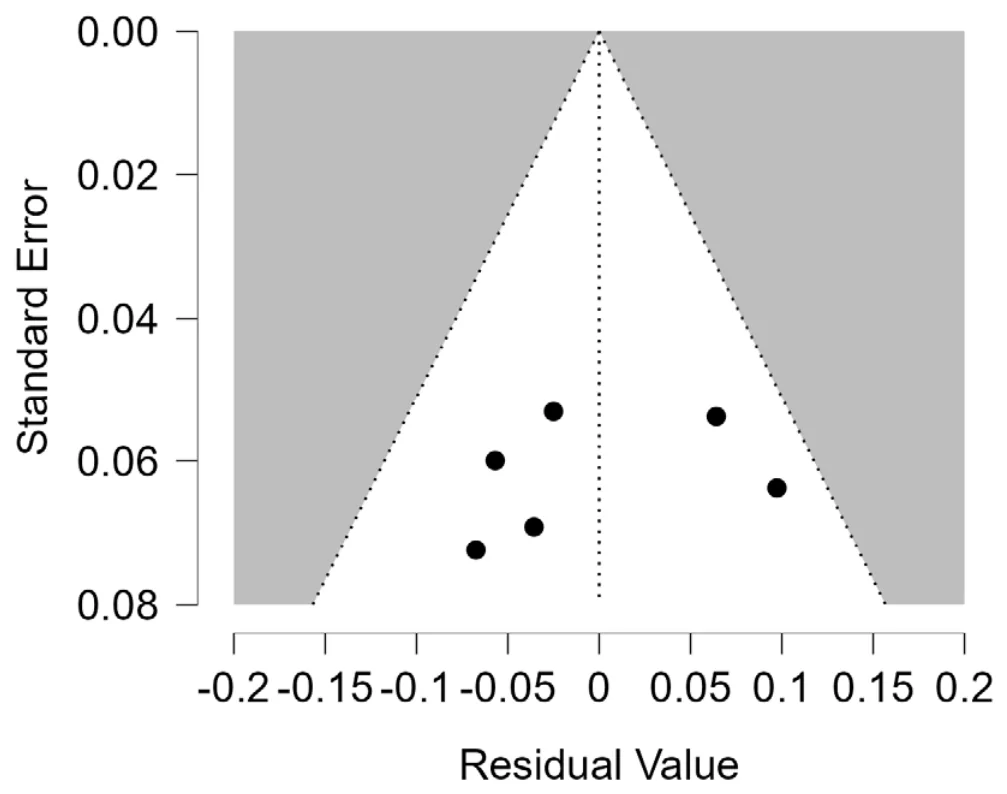
Residual Funnel Plot: Meta-Analysis of Work Overload and Turnover Intention. Note. Residual funnel plot for Work Overload and Turnover Intention. Vertical axis shows standard error (precision); horizontal axis displays residual effect sizes centered at zero. Dashed lines outline pseudo-95% confidence boundaries.

**Figure 8:**
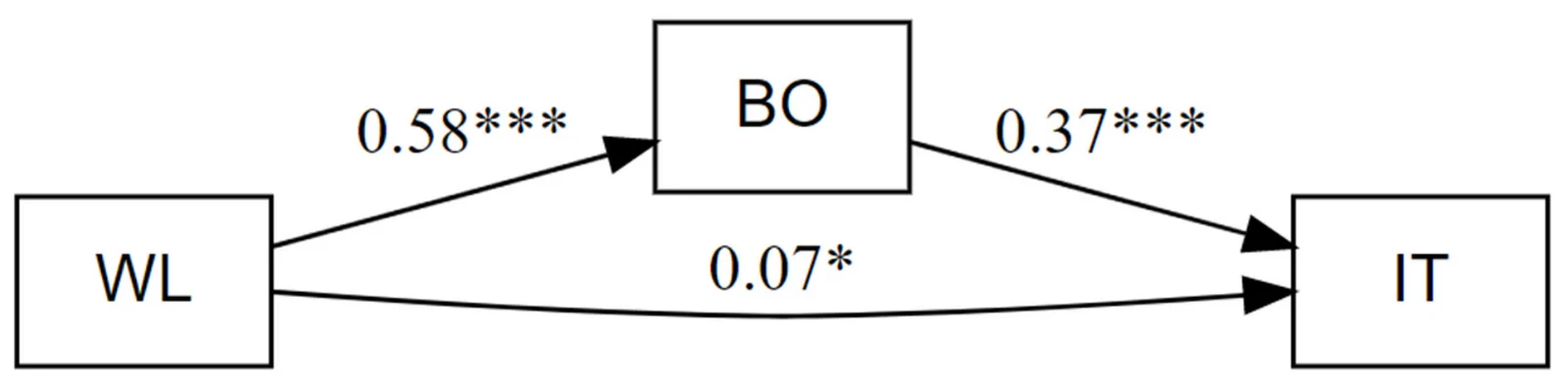
Meta-Analytic Mediation Model: Workload, Turnover Intention, and Burnout. Note: Exploratory structural model linking Workload (WL), Burnout (BO), and Turnover Intention (IT). Single-headed arrows represent the directional specification of regression paths within the model and do not imply causal relationships. * *p* < 0.05, *** *p* < 0.001.

**Table 2:** Narrative synthesis of evidence by construct, study design, measurement approach, direction and magnitude of associations, and methodological limitations.

Construct/Outcome	k	Study Design	Measurement Type	Instruments/Data Sources	Direction and Magnitude of Associations	Evidence Consistency	Main Methodological Limitations
Work overload	18	Cross-sectional (n = 15); longitudinal (n = 3)	Self-reported	COPSOQ, JCQ, SIMPH, NSS, ad hoc workload scales	Positive associations with burnout (r = 0.31–0.72; β = 0.25–0.60) and turnover intention (r = 0.06–0.37)	Consistent (+)	Potential common method variance; limited use of objective staffing and workload indicators
Mental workload	7	Cross-sectional (n = 6); longitudinal (n = 1)	Self-reported	COPSOQ (I, II, III), NASA-TLX	No direct correlation with exhaustion in one study (r = −0.02); weak positive association with job satisfaction (r = 0.12); adverse associations reported under low job control	Mixed/inconsistent	Conceptual overlap with quantitative workload; limited standardization of measurement
Work-related fatigue	5	Cross-sectional (n = 3); longitudinal (n = 2)	Self-reported	3D-WFI, SMBM/SMBQ, OLBI (exhaustion)	Positive associations with job demands and burnout-related outcomes (β = 0.21–0.44; r = 0.34–0.56)	Consistent (+)	Limited use of physiological measures or ecological momentary assessments during work shifts
Absenteeism (self-reported)	4	Cross-sectional (n = 3); longitudinal (n = 1)	Self-reported	Retrospective sickness absence reports, WHO-HPQ items	Higher proportions of prolonged sickness absence among workers with burnout (13.8% vs. 5.9%; *p* < 0.01)	Consistent (+)	Recall bias and potential social desirability bias
Absenteeism (administrative records)	3	Cross-sectional (n = 1); longitudinal (n = 1); quasi-experimental (n = 1)	Administrative records	Hospital personnel records, national registers (SLOSH)	Higher recorded sickness absence associated with job demands and insufficient resources (OR = 1.89; 95% CI [1.13, 3.13]); reductions following intervention (*p* < 0.001)	Consistent (+)	Difficulty distinguishing occupationally related sickness absence from absence due to non-occupational health conditions
Presenteeism	5	Cross-sectional (n = 3); longitudinal (n = 1); quasi-experimental (n = 1)	Primarily self-reported, with objective clinical performance indicators in one study	SPS-6, self-reported sickness presenteeism, medical error records	Positive associations with workload and burnout (β = 0.39–0.42); higher prevalence among workers with burnout (59.2% vs. 26.6%); association with medical errors (r = −0.28)	Consistent (+)	Predominantly subjective measurement; limited direct assessment of productivity losses
Actual turnover	3	Longitudinal/cohort (n = 3)	Administrative personnel records	Institutional personnel records and 12- to 18-month follow-up	Reported turnover rates of 0.6–19.7%; higher baseline job demands and exhaustion associated with subsequent turnover	Consistent (+)	Low event rates and potential attrition during follow-up

Note. k = number of studies; COPSOQ = Copenhagen Psychosocial Questionnaire; JCQ = Job Content Questionnaire; SIMPH = Social Inventory to Monitor Psychosocial Hazards; NSS = Nursing Stress Scale; NASA-TLX = NASA Task Load Index; 3D-WFI = Three-Dimensional Work Fatigue Inventory; SMBM/SMBQ = Shirom–Melamed Burnout Measure/Questionnaire; OLBI = Oldenburg Burnout Inventory; WHO-HPQ = World Health Organization Health and Work Performance Questionnaire; SPS-6 = Stanford Presenteeism Scale; SLOSH = Swedish Longitudinal Occupational Survey of Health.

**Table 3:** Summary of the random-effects meta-analytic models.

Model/Association	k	Pooled r	SE (z)	z-Score	95% CI (r)	95% PI (r)	Q (df = 5)	I^2^ (%)	Fail-Safe N
Work Overload → Burnout	6	0.58	0.02	37.03 ***	[0.55, 0.61]	[0.55, 0.61]	4.60	0.02%	2872 ***
Burnout → Turnover Intention	6	0.41	0.03	12.85 ***	[0.34, 0.48]	[0.24, 0.56]	17.78 **	70.21%	1262 ***
Work Overload → Turnover Intention	6	0.28	0.03	10.66 ***	[0.22, 0.35]	[0.15, 0.41]	10.72 †	53.61%	548 ***

Note. k = number of independent studies; r = back-transformed pooled Pearson correlation coefficient (r = tanh(z)); SE = standard error computed on Fisher’s z scale; CI = confidence interval; PI = prediction interval. † *p *<0.10, ** *p* < 0.01, *** *p* < 0.001.

**Table 4:** GRADE assessment of the certainty of evidence for the meta-analytic associations.

Association	Starting Certainty	Risk of Bias	Inconsistency	Indirectness	Imprecision	Publication Bias	Upgrading	Final Certainty
Work overload → Burnout	**Low**	Not additionally serious ^1^	Not serious ^5^	Not serious	Not serious	Inconclusive ^2^	None	**Low ⨁⨁◯◯**
Burnout → Turnover intention	**Low**	Not additionally serious ^1^	**Serious (−1) ^3^**	Not serious	Not serious	Inconclusive ^2^	None	**Very low ⨁◯◯◯**
Work overload → Turnover intention	**Low**	Not additionally serious ^1^	Not serious ^4^	Not serious	Not serious	Inconclusive ^2^	None	**Low ⨁⨁◯◯**

Note: ^1^ Certainty started at low because the meta-analytic evidence was derived from observational studies. The JBI assessments informed the risk-of-bias evaluation. Four of the five publications contributing to the meta-analyses identified confounding factors and reported strategies to address them, whereas one publication, contributing two independent samples, did not. However, all pooled associations were based on unadjusted zero-order correlations, and residual confounding could not be excluded. No additional downgrade for risk of bias was applied following consideration of these limitations. ^2^ Publication bias could not be reliably assessed because only six independent samples from five publications were available for each association. Funnel plots and statistical tests of asymmetry therefore had limited power. Although Begg and Egger tests did not detect statistically significant asymmetry, publication bias could not be excluded. ^3^ The burnout–turnover intention association was downgraded one level for serious inconsistency because substantial between-study heterogeneity was observed (I^2^ = 70.21%; Q(5) = 17.78). Although no individual estimate exceeded the Bonferroni-adjusted outlier threshold, the substantial heterogeneity warranted an additional downgrade. ^4^ Although moderate heterogeneity was observed for the work overload–turnover intention association (I^2^ = 53.61%), the direction of effects was consistent and the prediction interval remained above the null (PI = 0.15–0.41). Sensitivity analyses did not identify estimates with excessive influence on the pooled association. Therefore, inconsistency was not judged serious. ^5^ The work overload–burnout association showed the lowest heterogeneity among the three meta-analytic associations. Sensitivity analyses did not identify individual estimates with excessive influence on the pooled result, supporting the decision not to downgrade for inconsistency.

**Table 5:** Results of the Structural Meta-Analytic Mediation Model (TSSEM).

Parameter/Path	Estimate (β)	Standard Error (SE)	95% CI	z-Statistic	*p*-Value
*a* Path (Work Overload→ Burnout)	0.58	0.019	[0.544, 0.621]	29.89	<0.001
*b* Path (Burnout → Turnover Intention)	0.37	0.031	[0.312, 0.435]	11.88	<0.001
*c*′ Direct Path (Work Overload → Turnover Intention)	0.07	0.033	[0.001, 0.130]	1.99	0.047
Estimated Indirect Association (*a* × *b*)	0.22	0.019	[0.180, 0.256]	11.21	<0.001
Estimated Total Association (*c*′ + *a* × *b*)	0.28	0.038	[0.208, 0.358]	7.40	<0.001

*Note:* *N* = 1150 (sample harmonic mean). Estimated using Weighted Least Squares (WLS). Standardized coefficients (β). Proportion of total modeled association = 0.2175/0.2831 × 100 = 76.8%.

## Data Availability

No new data were created or analyzed in this study. Data sharing is not applicable to this article.

## Supplementary Materials

The following supporting information can be downloaded at: https://www.mdpi.com/article/10.3390/healthcare14203542/s1, Table S1: Study, variables, Pearson’s r, n, instruments, exclusion, reason for exclusion. Note S1. Methodological Rationale for Study Inclusion and Exclusion in Bivariate Meta-Analytic Syntheses. Note S2. Two-Stage Structural Equation Modeling (TSSEM) Procedure, Uncertainty Propagation, and Reproducibility.

## Appendix A

**Table A1:** Assessment of risk of bias in analytical cross-sectional studies using the JBI Critical Appraisal Tool for Analytical Cross-Sectional Studies.

Author and Year	Were the Sample Inclusion Criteria Clearly Defined?	Were Objective and Standardized Criteria Used to Measure the Condition?	Was Exposure Measured in a Valid and Reliable Manner?	Were the Outcomes Measured in a Valid and Reliable Manner?	Were Confounding Factors Identified?	Were Strategies Identified to Address Confounding Factors?	Was an Appropriate Statistical Analysis Used?	Were the Participants and the Study Context Described in Detail?
Albini et al. (2011)	Yes	Not applicable	Yes	Yes	Yes	Yes	Yes	Yes
Bruyneel et al. (2017)	Yes	Not applicable	Yes	Yes	Yes	Yes	Yes	Yes
Enea et al. (2024)	Yes	Not applicable	Yes	Yes	Yes	Yes	Yes	Yes
Gabel et al. (2016)	Yes	Not applicable	Yes	Yes	No	No	Yes	Yes
Hämmig (2018)	Yes	Not applicable	Yes	Yes	Yes	Yes	Yes	Yes
Hewko et al. (2015)	Unclear	Not applicable	Yes	Yes	Yes	Yes	Yes	Yes
Iguchi (2016)	Yes	Not applicable	Yes	Yes	Unclear	Unclear	Yes	Yes
Jourdain & Chênevert (2010)	Yes	Not applicable	Yes	Yes	Yes	Yes	Yes	Yes
Leep-Lazar et al. (2026)	Yes	Not applicable	Yes	Yes	Yes	Yes	Yes	Yes
Maniscalco et al. (2025)	Yes	Not applicable	Yes	Yes	Yes	Yes	Yes	Yes
Petersen et al. (2023)	Yes	Not applicable	Yes	Yes	Yes	Yes	Yes	Yes
Peterson et al. (2008)	Yes	Yes	Yes	Yes	No	No	Yes	Yes
Scanlan & Still (2019)	Yes	Not applicable	Unclear	Yes	Yes	Yes	Yes	Yes
Stordeur et al. (2007)	Yes	Yes	Yes	Yes	Unclear	Unclear	Yes	Yes
Tan et al. (2024)	Yes	Not applicable	Yes	Yes	Yes	Yes	Yes	Yes
Chen et al. (2019)	Yes	Not applicable	Yes	Yes	Yes	Yes	Yes	Yes
Chênevert et al. (2021)	Unclear	Not applicable	Yes	Yes	Yes	Yes	Yes	Yes
Luther et al. (2017)	Unclear	Not applicable	Yes	Yes	Yes	Yes	Yes	Yes
Peter et al. (2024)	Yes	Not applicable	Yes	Yes	Yes	Yes	Yes	Yes
Rotenstein et al. (2023)	Yes	Not applicable	Unclear	Yes	Yes	Yes	Yes	Yes
Barbon et al. (2026)	Yes	Not applicable	Yes	Yes	Yes	Yes	Yes	Yes
Van Bogaert et al. (2017)	Yes	Not applicable	Yes	Unclear	Unclear	Unclear	Yes	Yes
Moloney et al. (2018)	Yes	Not applicable	Yes	Yes	Unclear	Unclear	Yes	Yes
Odom-Forren et al. (2025)	Yes	Not applicable	Yes	Yes	Yes	Yes	Yes	Yes
Wong et al. (2024)	Yes	Not applicable	Yes	Yes	Unclear	Unclear	Yes	Yes
Xian et al. (2020)	Yes	Not applicable	Yes	Yes	Yes	Yes	Yes	Yes
Adriaenssens et al. (2017)	Yes	Not applicable	Yes	Yes	Yes	Yes	Yes	Yes
Vandenbroeck et al. (2017)	Yes	Yes	Yes	Yes	Unclear	Unclear	Yes	Yes

Note: Response categories: Yes, No, Unclear, and Not applicable.

**Table A2:** Assessment of the risk of bias in longitudinal studies using the JBI Critical Appraisal Tool for Cohort Studies.

Author and Year	Were the Two Groups Similar and Recruited from the Same Population?	Were Exposures Measured in a Similar Manner When Assigning Individuals to the Exposed and Unexposed Groups?	Was the Exposure Measured in a Valid and Reliable Manner?	Were Confounding Factors Identified?	Were Strategies Outlined to Address Confounding Factors?	Were the Groups/Participants Free of the Outcome at the Start of the Study (or at the Time of Exposure)?	Were the Outcomes Measured in a Valid and Reliable Manner?	Was the Follow-up Duration Reported, and was it Long Enough to Allow the Outcomes to Occur?	Was Follow-up Complete, and if not, Were the Reasons for Follow-up Losses Described and Explored?	Were Strategies Used to Address Incomplete Follow-up?
Adriaenssens et al. (2015)	Yes	Yes	Yes	Yes	Yes	No	Yes	Yes	Yes	No
Cheyroux et al. (2025)	Yes	Yes	Yes	Unclear	Unclear	No	Yes	Yes	Yes	Yes
Thapa et al. (2022)	Yes	Yes	Yes	Yes	Yes	No	Yes	Yes	Unclear	No
Van der Heijden et al. (2019)	Yes	Yes	Yes	Yes	Yes	No	Yes	Yes	No	No

Note: Response categories: Yes, No, Unclear, and Not applicable.

**Table A3:** Assessment of the risk of bias in quasi-experimental studies using the JBI Critical Appraisal Checklist for Quasi-Experimental Studies.

Author and Year	Is It Clear in the Study What the “Cause” Is and What the “Effect” Is (i.e., There Is no Confusion Regarding Which Variable Occurs First)?	Was There a Control Group?	Were the Participants Included in the Comparisons Similar?	Did the Participants Included in the Comparisons Receive Similar Treatment/Care, Apart from the Exposure or Intervention of Interest?	Were There Multiple Measurements of the Outcome, Both Before and After the Intervention or Exposure?	Were the Outcomes of the Participants Included in the Comparisons Measured in the Same Way?	Were the Outcomes Measured Reliably?	Was Follow-up Complete, and If Not, Were Differences Between Groups Regarding Follow-up Adequately Described and Analyzed?	Was an Appropriate Statistical Analysis Used?
Rickard et al. (2012)	yes	no	yes	unclear	no	yes	yes	yes	yes
Qiao et al. (2026)	yes	yes	yes	yes	no	yes	yes	yes	yes

Note: Response categories: Yes, No, Unclear, and Not applicable.

**Table A4:** Reports excluded after full-text assessment and reasons for exclusion.

Excluded Report	Reason for Exclusion
Opie et al. (2010) [66]	Ineligible occupational outcome. The study assessed occupational stress/workload and burnout-related variables but did not assess an eligible withdrawal outcome (turnover intention, actual turnover, absenteeism, or sick leave).
Wen et al. (2026) [67]	Ineligible exposure. The study assessed burnout and turnover intention but did not assess mental workload or work overload as an exposure.
Sulca (2021) [68]	Ineligible exposure. The study assessed work fatigue and absenteeism but did not empirically assess mental workload or work overload as an exposure.
Kwon et al. (2021) [69]	Ineligible exposure. The study assessed emotional labor, burnout, and turnover intention, but did not assess mental workload or work overload as defined in the review eligibility criteria.
