# Supplementary material for: Work Demands, Burnout, and Withdrawal Behaviors Among Healthcare Workers: A Systematic Review and Meta-Analysis
Source: Healthcare (Basel). 2026 Oct 21;14(20):3542. doi: 10.3390/healthcare14203542 (PMC13704590; doi:10.3390/healthcare14203542)
Supplement: Supplementary file 1 [file healthcare-14-03542-s001.zip › healthcare-4598754-supplementary.pdf]

Supplementary Note S1. Methodological Rationale for Study Inclusion and Exclusion in Bivariate Meta-Analytic Syntheses

A total of 34 primary studies met the criteria for inclusion in the overall systematic review. However, only 5 publications yielding 6 independent samples provided fully comparable data to be incorporated into quantitative bivariate meta-analyses and the exploratory two-stage structural equation modeling (TSSEM).

To ensure statistical rigor and comparability across effect sizes, eligibility for quantitative meta-analysis required primary studies to fulfill all the following criteria:

- Explicit measurement of quantitative work overload or quantitative demands.
- Assessment of burnout or its primary dimension, emotional exhaustion.
- Assessment of organizational turnover intention, distinguished from actual turnover, sickness absence, or presenteeism.
- Availability of zero-order Pearson correlation coefficients (r) alongside corresponding sample sizes (N) for the exact variable pairs.
- Verifiable sample independence.

The remaining 29 studies (or specific variable pairs within included publications) could not be included in the quantitative syntheses due to specific methodological and statistical reasons:

1. **Non-Comparable Statistical Effect Estimators ($k = 21$ studies)**:
    - **Structural Equation Modeling (SEM/PLS) Path Coefficients**: Studies reporting only standardized path coefficients derived from multivariate structural models (e.g., Bruyneel et al., 2017; Iguchi, 2016; Moloney et al., 2018; Van Bogaert et al., 2017; Vandenbroeck et al., 2017) were excluded because path coefficients reflect partialized relationships adjusted for other model covariates and cannot be mathematically treated as zero-order bivariate correlations (r).
    - **Regression Beta/B Coefficients**: Studies reporting multiple linear regression coefficients (β or B) without providing zero-order correlation matrices (e.g., Hämmig, 2018; Leep-Lazar et al., 2026; Peter et al., 2024; Tan et al., 2024) were excluded because partial regression weights depend on model specifications and are not directly comparable to bivariate correlations.
    - **Odds Ratios (OR) and Risk Ratios (RR)**: Studies presenting binary or multinomial logistic regression outcomes (e.g., Chen et al., 2019; Enea et al., 2024; Maniscalco et al., 2025; Odom-Forren et al., 2025; Rotenstein et al., 2023; Thapa et al., 2022) were excluded

because converting ORs to Pearson's r requires distributional assumptions that introduce artificial bias when underlying variables are continuous scales.

- **Non-Parametric Correlation Metrics**: Studies reporting Spearman's rank correlations (ρ) (e.g., Wong et al., 2024) were excluded to maintain estimator homogeneity and avoid mixing parametric and non-parametric effect sizes.
- **Group Mean Comparisons and Univariate Statistics**: Studies presenting findings strictly via mean comparisons, t-tests, ANOVA, or intergroup discriminant analyses (e.g., Hewko et al., 2015; Luther et al., 2017; Peterson et al., 2008; Rickard et al., 2012; Stordeur et al., 2007) lacked continuous association metrics eligible for pooling.

2. **Construct Divergence and Ineligible Outcome Variables ($k = 5$ studies)**:

   - Studies that did not measure organizational turnover intention (Y), focusing instead exclusively on absenteeism, presenteeism, sick leave days, or clinical performance indicators (e.g., Albini et al., 2011; Petersen et al., 2023; Peterson et al., 2008; Qiao et al., 2026; Xian et al., 2020), were excluded from quantitative synthesis to prevent outcome contamination.

3. **Non-Comparable Analytic Designs ($k = 3$ studies)**:

   - **Person-Centered Approaches**: Studies employing Latent Profile Analysis or Growth Mixture Modeling (GMM) (e.g., Cheyroux et al., 2025) were excluded because profile-based transition probabilities cannot be converted into variable-centered correlation matrices.
   - **Longitudinal/Intervention Datasets without Full Baseline Correlation Matrices**: Longitudinal panel studies or intervention evaluations (e.g., Adriaenssens et al., 2015; Van der Heijden et al., 2019) that did not report the complete 3×3 cross-sectional Pearson correlation matrix among workload, burnout, and turnover intention at baseline were excluded.

Table S1 provides the complete overview of all evaluated primary studies, detailed variable pairs, reported correlation metrics, sample sizes, and specific exclusion justifications.

Table S1: Study, variables, Pearson's r, n, instruments, exclusion, reason for exclusion

Authors	year	pair	variable_1	variable_2	r	N_pair	Instruments Used	Exclude?	Reason for Exclusion
Adriaenssens et al.	2015	X_M	X: workload/demands	M: burnout/exhaustion	0,3	170	X: LQWQ-N / M: MBI (EE)	Yes	longitudinal
Adriaenssens et al.	2015	M_Y	M: burnout/exhaustion	Y: turnover intention	-0,09	170	M: MBI (EE) / Y: LQWQ-N	Yes	longitudinal
Adriaenssens et al.	2015	X_Y	X: workload/demands	Y: turnover intention	-0,04	170	X: LQWQ-N / Y: LQWQ-N	Yes	longitudinal
Adriaenssens et al.	2017	X_M	X: workload/demands	M: burnout (global)	0,324	318	X: LQWQ (PreYesón) / M: MBI-HSS	Yes	Contradictory Relations
Adriaenssens et al.	2017	M_Y	M: burnout (global)	Y: turnover intention	0,42	318	M: MBI-HSS / Y: LQWQ-N	Yes	Contradictory Relations
Adriaenssens et al.	2017	X_Y	X: workload/demands	Y: turnover intention	0,138	318	X: LQWQ (PreYesón) / Y: LQWQ-N	Yes	Contradictory Relations
Albini et al.	2011	X_M	X: workload/demands	M: burnout/exhaustion	0,255	230	X: JCQ (JD) / M: MBI (EE)	Yes	Does Not Measure Y
Albini et al.	2011	M_Y	M: burnout/exhaustion	Y: turnover intention	N/A	230	M: MBI (EE) / Y: No medida	Yes	Does Not Measure Y
Albini et al.	2011	X_Y	X: workload/demands	Y: turnover intention	N/A	230	X: JCQ (JD) / Y: No medida	Yes	Does Not Measure Y
Barbon et al.	2026	X_M	X: workload/demands	M: burnout/exhaustion	0,573	790	X: YEST (Demand) / M: COPSOQ (EE)	No	
Barbon et al.	2026	M_Y	M: burnout/exhaustion	Y: turnover intention	0,503	772	M: COPSOQ (EE) / Y: Kelloway et al.	No	
Barbon et al.	2026	X_Y	X: workload/demands	Y: turnover intention	0,341	771	X: YEST (Demand) / Y: Kelloway et al.	No	
Bruyneel et al.	2017	X_M	X: workload/demands	M: burnout/exhaustion	N/A	294	X: LQWQ-N / M: MBI-HSS	Yes	Report only path coefficients (SEM)
Bruyneel et al.	2017	M_Y	M: burnout/exhaustion	Y: turnover intention	N/A	294	M: MBI-HSS / Y: LQWQ-N	Yes	Report only path coefficients (SEM)
Bruyneel et al.	2017	X_Y	X: workload/demands	Y: turnover intention	N/A	294	X: LQWQ-N / Y: LQWQ-N	Yes	Report only path coefficients (SEM)
Chen et al.	2019	X_M	X: workload/demands	M: burnout/exhaustion	N/A	1409	X: ADPNR / M: C-CBI (Personal)	Yes	Results on odds ratios and mediation.

Chen et al.	2019	M_Y	M: burnout/exhaustion	Y: turnover intention	N/A	1409	M: C-CBI (Personal) / Y: 2-item scale	Yes	Results on odds ratios and mediation.
Chen et al.	2019	X_Y	X: workload/demands	Y: turnover intention	N/A	1409	X: ADPNR / Y: 2-item scale	Yes	Results on odds ratios and mediation.
Chênevert et al.	2021	X_M	X: workload/demands	M: burnout/exhaustion	0,61	407	X: Caplan et al. / M: MBI-HSS (EE)	No	
Chênevert et al.	2021	M_Y	M: burnout/exhaustion	Y: turnover intention	0,44	407	M: MBI-HSS (EE) / Y: Meyer et al.	No	
Chênevert et al.	2021	X_Y	X: workload/demands	Y: turnover intention	0,37	407	X: Caplan et al. / Y: Meyer et al.	No	
Cheyroux et al.	2025	X_M	X: workload/demands	M: burnout/exhaustion	N/A	622	X: Lequeurre et al. / M: 3D-WFI (Fatiga)	Yes	Latent Profile AnalyYess (GMM)
Cheyroux et al.	2025	M_Y	M: burnout/exhaustion	Y: turnover intention	N/A	622	M: 3D-WFI (Fatiga) / Y: Bardach et al.	Yes	Latent Profile AnalyYess (GMM)
Cheyroux et al.	2025	X_Y	X: workload/demands	Y: turnover intention	N/A	622	X: Lequeurre et al. / Y: Bardach et al.	Yes	Latent Profile AnalyYess (GMM)
Enea et al.	2024	X_M	X: workload/demands	M: burnout/exhaustion	N/A	1350	X: Sorra et al. / M: MBI (EE/DP)	Yes	Report only adjusted odds ratios.
Enea et al.	2024	M_Y	M: burnout/exhaustion	Y: turnover intention	N/A	1350	M: MBI (EE/DP) / Y: Kaihlanen et al.	Yes	Report only adjusted odds ratios.
Enea et al.	2024	X_Y	X: workload/demands	Y: turnover intention	N/A	1350	X: Sorra et al. / Y: Kaihlanen et al.	Yes	Report only adjusted odds ratios.
Gabel Shemueli et al. (UY)	2016	X_M	X: workload/demands	M: burnout/exhaustion	0,57	316	X: Karasek et al. / M: SMBM / Y: Arsenault et al.	No	
Gabel Shemueli et al. (UY)	2016	M_Y	M: burnout/exhaustion	Y: turnover intention	0,35	316	X: Karasek et al. / M: SMBM / Y: Arsenault et al.	No	
Gabel Shemueli et al. (UY)	2016	X_Y	X: workload/demands	Y: turnover intention	0,25	316	X: Karasek et al. / M: SMBM / Y: Arsenault et al.	No	
Gabel Shemueli et al. (ES)	2016	X_M	X: workload/demands	M: burnout/exhaustion	0,59	502	X: Karasek et al. / M: SMBM / Y: Arsenault et al.	No	
Gabel Shemueli et al. (ES)	2016	M_Y	M: burnout/exhaustion	Y: turnover intention	0,32	502	X: Karasek et al. / M: SMBM / Y: Arsenault et al.	No	
Gabel Shemueli et al. (ES)	2016	X_Y	X: workload/demands	Y: turnover intention	0,23	502	X: Karasek et al. / M: SMBM / Y: Arsenault et al.	No	

Hämmig	2018	X_M	X: workload/demands	M: burnout/exhaustion	N/A	1441	X: COPSOQ / M: CBI (Personal)	Yes	It reports beta coefficients and odds ratios.
Hämmig	2018	M_Y	M: burnout/exhaustion	Y: turnover intention	N/A	1441	M: CBI (Personal) / Y: COPSOQ (NEXT)	Yes	It reports beta coefficients and odds ratios.
Hämmig	2018	X_Y	X: workload/demands	Y: turnover intention	N/A	1441	X: COPSOQ / Y: COPSOQ (NEXT)	Yes	It reports beta coefficients and odds ratios.
Hewko et al.	2015	X_M	X: workload/resources	M: burnout/exhaustion	N/A	95	X: Factors ad hoc / M: MBI (EE)	Yes	Comparison of averages and rankings.
Hewko et al.	2015	M_Y	M: burnout/exhaustion	Y: turnover intention	N/A	95	M: MBI (EE) / Y: Category (stay/leave)	Yes	Comparison of averages and rankings.
Hewko et al.	2015	X_Y	X: workload/resources	Y: turnover intention	N/A	95	X: Factors ad hoc / Y: Category (stay/leave)	Yes	Comparison of averages and rankings.
Iguchi	2016	X_M	X: workload/demands	M: burnout/exhaustion	N/A	1766	X: JD-R Questionnaire / M: Scale based on the MBI	Yes	Report path coefficients (SEM)
Iguchi	2016	M_Y	M: burnout/exhaustion	Y: turnover intention	N/A	1766	M: MBI-based scale / Y: 3-item scale	Yes	Report path coefficients (SEM)
Iguchi	2016	X_Y	X: workload/demands	Y: turnover intention	N/A	1766	X: JD-R Questionnaire / Y: 3-item scale	Yes	Report path coefficients (SEM)
Jourdain & Chênevert	2010	X_M	X: workload/demands	M: burnout/exhaustion	0,6	818	X: Caplan et al. / M: MBI-HSS (EE)	No	
Jourdain & Chênevert	2010	M_Y	M: burnout/exhaustion	Y: turnover intention	0,41	818	M: MBI-HSS (EE) / Y: Meyer et al.	No	
Jourdain & Chênevert	2010	X_Y	X: workload/demands	Y: turnover intention	0,26	818	X: Caplan et al. / Y: Meyer et al.	No	
Leep-Lazar et al.	2026	X_M	X: workload/demands	M: burnout/exhaustion	N/A	770	X: COPSOQ / M: CBI	Yes	Reports regresYeson beta coefficients.
Leep-Lazar et al.	2026	M_Y	M: burnout/exhaustion	Y: turnover intention	N/A	770	M: CBI / Y: NSSRN (adapted)	Yes	Reports regresYeson beta coefficients.
Leep-Lazar et al.	2026	X_Y	X: workload/demands	Y: turnover intention	N/A	770	X: COPSOQ / Y: NSSRN (adapted)	Yes	Reports regresYeson beta coefficients.
Luther et al.	2017	X_M	X: workload/overtime	M: burnout/exhaustion	N/A	182	X: Overtime / M: MBI	Yes	A comparison of means is reported
Luther et al.	2017	M_Y	M: burnout/exhaustion	Y: turnover intention	N/A	182	M: MBI / Y: Yesngle self-reported item	Yes	A comparison of means is reported

Luther et al.	2017	X_Y	X: workload/overtime	Y: turnover intention	N/A	182	X: Overtime / Y: Yesngle self-reported item	Yes	A comparison of means is reported
Maniscalco et al.	2025	X_M	X: workload/demands	M: burnout/exhaustion	N/A	375	X: METEOR (Demands) / M: MBI	Yes	Report only adjusted odds ratios.
Maniscalco et al.	2025	M_Y	M: burnout/exhaustion	Y: turnover intention	N/A	375	M: MBI / Y: Kaihlanen et al.	Yes	Report only adjusted odds ratios.
Maniscalco et al.	2025	X_Y	X: workload/demands	Y: turnover intention	N/A	375	X: METEOR (Demands) / Y: Kaihlanen et al.	Yes	Report only adjusted odds ratios.
Moloney et al.	2018	X_M	X: workload/demands	M: burnout	N/A	2876	X: COPSOQ / M: BMS-10	Yes	Report path coefficients (SEM)
Moloney et al.	2018	M_Y	M: burnout	Y: turnover intention	N/A	2876	M: BMS-10 / Y: Dotson et al.	Yes	Report path coefficients (SEM)
Moloney et al.	2018	X_Y	X: workload/demands	Y: turnover intention	N/A	2876	X: COPSOQ / Y: Dotson et al.	Yes	Report path coefficients (SEM)
Odom-Forren et al.	2025	X_M	X: workload/demands	M: burnout	N/A	1436	X: NSS / M: ProQOL (Burnout)	Yes	Reports only odds ratios (logistic regresYeson).
Odom-Forren et al.	2025	M_Y	M: burnout	Y: intent to stay	N/A	1436	M: ProQOL / Y: Bacon & Stewart	Yes	Reports only odds ratios (logistic regresYeson).
Odom-Forren et al.	2025	X_Y	X: workload/demands	Y: intent to stay	N/A	1436	X: NSS / Y: Bacon & Stewart	Yes	Reports only odds ratios (logistic regresYeson).
Peter et al.	2024	X_M	X: workload/demands	M: burnout	N/A	19340	X: STRAIN (COPSOQ) / M: STRAIN (Burnout)	Yes	Reports beta coefficients (multilevel).
Peter et al.	2024	M_Y	M: burnout	Y: turnover intention	N/A	19340	M: STRAIN (Burnout) / Y: STRAIN (COPSOQ)	Yes	Reports beta coefficients (multilevel).
Peter et al.	2024	X_Y	X: workload/demands	Y: turnover intention	N/A	19340	X: STRAIN (COPSOQ) / Y: STRAIN (COPSOQ)	Yes	Reports beta coefficients (multilevel).
Petersen et al.	2023	X_M	X: workload/demands	M: emotional exhaustion	N/A	585	X: BIBB/BAuA / M: OLBI (EE)	Yes	It does not measure turnover intention; it uses odds ratios.
Petersen et al.	2023	M_Y	M: emotional exhaustion	Y: turnover intention	N/A	585	M: OLBI (EE) / Y: Not measured	Yes	It does not measure turnover intention; it uses odds ratios.
Petersen et al.	2023	X_Y	X: workload/demands	Y: turnover intention	N/A	585	X: BIBB/BAuA / Y: Not measured	Yes	It does not measure turnover intention; it uses odds ratios.
Peterson et al.	2008	X_M	X: workload/demands	M: emotional exhaustion	N/A	3616	X: QPS Nordic / M: OLBI (EE)	Yes	Intergroup discriminant analyYess.

Peterson et al.	2008	M_Y	M: emotional exhaustion	Y: turnover intention	N/A	3616	M: OLBI (EE) / Y: Not measured	Yes	Intergroup discriminant analyYess.
Peterson et al.	2008	X_Y	X: workload/demands	Y: turnover intention	N/A	3616	X: QPS Nordic / Y: Not measured	Yes	Intergroup discriminant analyYess.
Qiao et al.	2026	X_M	X: workload/demands	M: emotional exhaustion	N/A	600	X: Indicadores laborales / M: MBI (EE)	Yes	It does not measure turnover intention; it uses beta coefficients.
Qiao et al.	2026	M_Y	M: emotional exhaustion	Y: turnover intention	N/A	600	M: MBI (EE) / Y: Not measured	Yes	It does not measure turnover intention; it uses beta coefficients.
Qiao et al.	2026	X_Y	X: workload/demands	Y: turnover intention	N/A	600	X: Indicadores laborales / Y: Not measured	Yes	It does not measure turnover intention; it uses beta coefficients.
Rickard et al.	2012	X_M	X: workload/demands	M: burnout/exhaustion	N/A	306	X: Herramienta de carga / M: MBI	Yes	Intervention evaluation (comparison of means).
Rickard et al.	2012	M_Y	M: burnout/exhaustion	Y: turnover intention	N/A	306	M: MBI / Y: Turnover Data	Yes	Intervention evaluation (comparison of means).
Rickard et al.	2012	X_Y	X: workload/demands	Y: turnover intention	N/A	306	X: Herramienta de carga / Y: Turnover Data	Yes	Intervention evaluation (comparison of means).
Rotenstein et al.	2023	X_M	X: workload/overload	M: burnout	N/A	43026	X: Yesngle item / M: Yesngle item	Yes	Reports Relative Risks and Odds Ratios.
Rotenstein et al.	2023	M_Y	M: burnout	Y: turnover intention	N/A	43026	M: Yesngle item / Y: Yesngle item	Yes	Reports Relative Risks and Odds Ratios.
Rotenstein et al.	2023	X_Y	X: workload/overload	Y: turnover intention	N/A	43026	X: Yesngle item / Y: Yesngle item	Yes	Reports Relative Risks and Odds Ratios.
Scanlan & Still	2019	X_M	X: job demands	M: exhaustion	0,51	277	X: JD-RQ / M: OLBI (Exhaustion)	No	
Scanlan & Still	2019	M_Y	M: exhaustion	Y: turnover intention	0,42	277	M: OLBI / Y: Ali Shah et al.	No	
Scanlan & Still	2019	X_Y	X: job demands	Y: turnover intention	0,22	277	X: JD-RQ / Y: Ali Shah et al.	No	
Stordeur et al.	2007	X_M	X: workload/demands	M: burnout/exhaustion	N/A	1175	X: Escalas NEXT / M: NEXT (Exhaustion)	Yes	Comparison of means (t-test).
Stordeur et al.	2007	M_Y	M: burnout/exhaustion	Y: turnover intention	N/A	1175	M: NEXT / Y: NEXT (Intention)	Yes	Comparison of means (t-test).

Stordeur et al.	2007	X_Y	X: workload/demands	Y: turnover intention	N/A	1175	X: Escalas NEXT / Y: NEXT (Intention)	Yes	Comparison of means (t-test).
Tan et al.	2024	X_M	X: job demands	M: burnout	N/A	60	X: COPSOQ III / M: ProQOL5	Yes	Reports regresYeson coefficients (B).
Tan et al.	2024	M_Y	M: burnout	Y: turnover intention	N/A	60	M: ProQOL5 / Y: TIS-6	Yes	Reports regresYeson coefficients (B).
Tan et al.	2024	X_Y	X: job demands	Y: turnover intention	N/A	60	X: COPSOQ III / Y: TIS-6	Yes	Reports regresYeson coefficients (B).
Thapa et al.	2022	X_M	X: workload/demands	M: burnout	N/A	1064	X: COPSOQ II / M: Yesngle item	Yes	It does not measure turnover intention; it uses odds ratios.
Thapa et al.	2022	M_Y	M: burnout	Y: turnover intention	N/A	1064	M: Yesngle item / Y: Not measured	Yes	It does not measure turnover intention; it uses odds ratios.
Thapa et al.	2022	X_Y	X: workload/demands	Y: turnover intention	N/A	1064	X: COPSOQ II / Y: Not measured	Yes	It does not measure turnover intention; it uses odds ratios.
Van Bogaert et al.	2017	X_M	X: workload/demands	M: burnout	N/A	N/A	X: FIT / M: MBI-HSS	Yes	Reports path coefficients (SEM).
Van Bogaert et al.	2017	M_Y	M: burnout	Y: job outcomes	N/A	N/A	M: MBI-HSS / Y: Pes-NWI (adapted)	Yes	Reports path coefficients (SEM).
Van Bogaert et al.	2017	X_Y	X: workload/demands	Y: job outcomes	N/A	N/A	X: FIT / Y: Pes-NWI (adapted)	Yes	Reports path coefficients (SEM).
Van der Heijden et al.	2019	X_M	X: perceived effort	M: burnout	0,34	1187	X: Based on ERI / M: CBI	Yes	longitudinal
Van der Heijden et al.	2019	M_Y	M: burnout	Y: turnover intention	0,36	1187	M: CBI / Y: Intention Scale	Yes	longitudinal
Van der Heijden et al.	2019	X_Y	X: perceived effort	Y: turnover intention	0,06	1187	X: Based on ERI / Y: Intention Scale	Yes	longitudinal
Vandenbroeck et al.	2017	X_M	X: workload	M: emotional exhaustion	N/A	5700	X: YESMPH / M: UBOS (MBI)	Yes	Reports path coefficients (SEM).
Vandenbroeck et al.	2017	M_Y	M: emotional exhaustion	Y: turnover intention	N/A	5700	M: UBOS / Y: Ad hoc items	Yes	Reports path coefficients (SEM).
Vandenbroeck et al.	2017	X_Y	X: workload	Y: turnover intention	N/A	5700	X: YESMPH / Y: Ad hoc items	Yes	Reports path coefficients (SEM).
Wong et al.	2024	X_M	X: work overload	M: burnout	0,495	502	X: Load scale / M: MBI	Yes	Reports spearman rho

Wong et al.	2024	M_Y	M: burnout	Y: turnover intention (org)	0,557	502	M: MBI / Y: Yamaguchi et al.	Yes	Reports spearman rho
Wong et al.	2024	M_Y	M: burnout	Y: turnover intention (prof)	0,454	502	M: MBI / Y: Yamaguchi et al.	Yes	Measures profesYesonal turnover intention
Wong et al.	2024	X_Y	X: work overload	Y: turnover intention (org)	0,327	502	X: Load scale / Y: Yamaguchi et al.	Yes	Reports spearman rho
Wong et al.	2024	X_Y	X: work overload	Y: turnover intention (prof)	0,339	502	X: Load scale / Y: Yamaguchi et al.	Yes	Measures profesYesonal turnover intention
Xian et al.	2020	X_M	X: job demands	M: burnout (weighted)	N/A	366	X: Li et al. Scale / M: MBI-GS	Yes	It does not measure turnover intention (Variable Y).
Xian et al.	2020	M_Y	M: burnout (weighted)	Y: turnover intention	N/A	366	M: MBI-GS / Y: Not measured	Yes	It does not measure turnover intention (Variable Y).
Xian et al.	2020	X_Y	X: job demands	Y: turnover intention	N/A	366	X: Escala de Li et al. / Y: Not measured	Yes	It does not measure turnover intention (Variable Y).

Results of the Meta-analysis on Work Overload and Burnout (JASP and JAMOVI)

Classical Meta-Analysis

Meta-Analytic Tests

	Test	p
Heterogeneity	$Q_e(5) = 4.60$	.467
Pooled effect	$t(5) = 38.63$	< .001

Meta-Analytic Estimates

		95% CI		95% PI	
	Estimate	Lower	Upper	Lower	Upper
Pooled effect	0.5824	0.5523	0.6109	0.5523	0.6109
τ	6.523×10^{-4}	0.000	0.1143		
τ^2	4.254×10^{-7}	0.000	0.01306		
I^2	0.02116	0.000	86.66		

Note. The pooled effect is transformed using Fisher's z to r transformation. For nonlinear transformations, the transformed estimate is interpreted as a median on the transformed scale.

Forest Plot

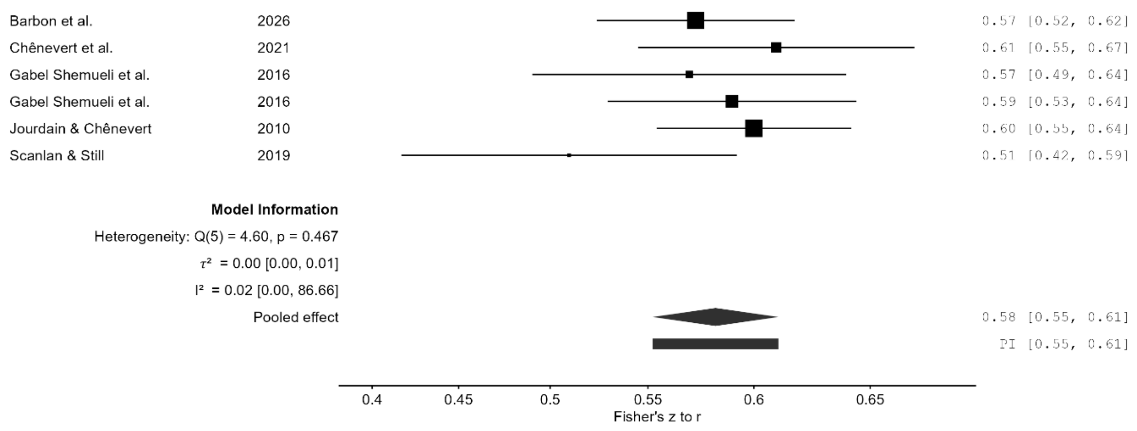

Casewise Diagnostics Table

				Leave One Out				
Standardized Residual	DFFITS	Cook's Distance	Covariance ratio	τ	τ^2	Q_e	Hat	Weight
-0.4367	-0.2553	0.07774	1.600	1.122×10^{-4}	1.258×10^{-8}	4.387	0.2545	25.45
0.9553	0.3703	0.1396	1.171	2.715×10^{-4}	7.369×10^{-8}	3.743	0.1307	13.07
-0.3269	-0.1098	0.01467	1.355	4.024×10^{-4}	1.620×10^{-7}	4.477	0.1012	10.12
0.2665	0.1169	0.01677	1.464	2.240×10^{-4}	5.016×10^{-8}	4.516	0.1614	16.14
0.9259	0.5541	0.3166	1.399	0.002142	4.588×10^{-6}	3.784	0.2636	26.36
-3.043	-0.9488	0.3395	0.4138	0.000	0.000	1.387	0.08863	8.863

Residual Funnel Plot

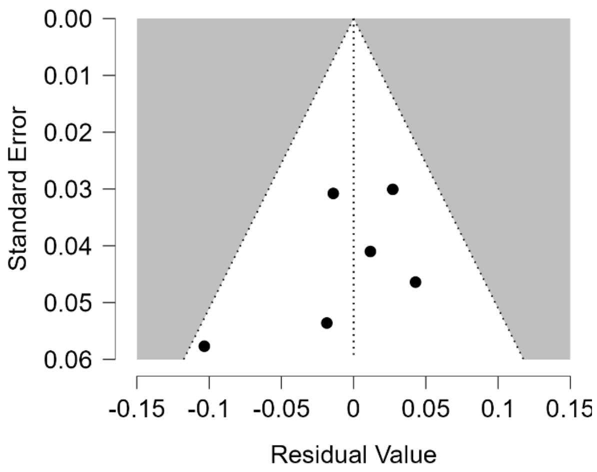

Publication Bias Assessment

Test Name	value	p
Fail-Safe N	2872.000	<.001
Begg and Mazumdar Rank Correlation	-0.333	.469
Egger's Regression	-1.068	.286
Trim and Fill Number of Studies	0.000	.

Nota. Fail-safe N Calculation Using the Rosenthal Approach

Results of the Meta-analysis on Burnout and Turnover Intention (JASP and JAMOVI)

Classical Meta-Analysis

Model Summary

Meta-Analytic Tests

	Test	p
Heterogeneity	$Q_e(5) = 17.78$	.003
Pooled effect	$t(5) = 13.25$	< .001

Meta-Analytic Estimates

		95% CI		95% PI	
	Estimate	Lower	Upper	Lower	Upper
Pooled effect	0.4117	0.3388	0.4796	0.2364	0.5610
τ	0.06899	0.02581	0.1884		
τ^2	0.004760	6.662×10^{-4}	0.03548		
I^2	70.21	24.80	94.61		

Note. The pooled effect is transformed using Fisher's z to r transformation. For nonlinear transformations, the transformed estimate is interpreted as a median on the transformed scale.

Forest Plot

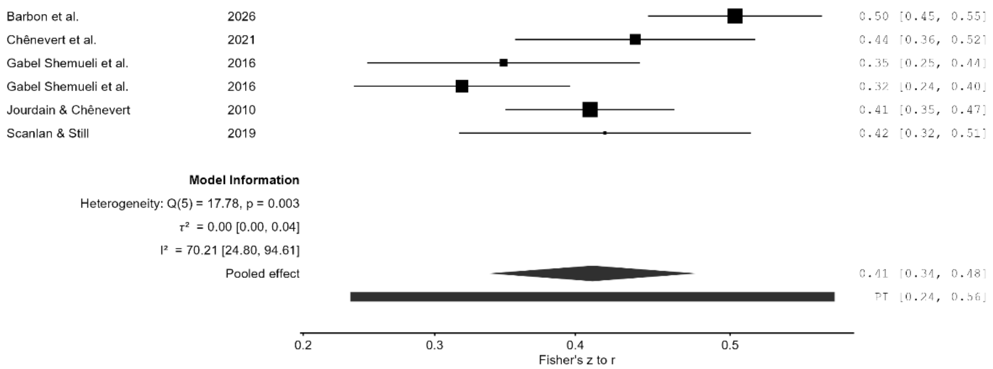

Casewise Diagnostics Table

				Leave One Out				
Standardized Residual	DFFITS	Cook's Distance	Covariance ratio	τ	τ^2	Q_e	Hat	Weight
2.520	1.239	0.6776	0.6398	0.03603	0.001298	6.169	0.1913	19.13
0.4289	0.2040	0.04898	1.415	0.07755	0.006013	17.48	0.1602	16.02
-0.8847	-0.3636	0.1373	1.223	0.07084	0.005018	15.46	0.1457	14.57
-1.743	-0.8436	0.5122	0.8710	0.05359	0.002872	9.860	0.1714	17.14
-0.01702	0.01080	1.489×10^{-4}	1.525	0.08068	0.006510	17.63	0.1936	19.36
0.1179	0.06416	0.004940	1.442	0.07796	0.006077	17.78	0.1378	13.78

Residual Funnel Plot

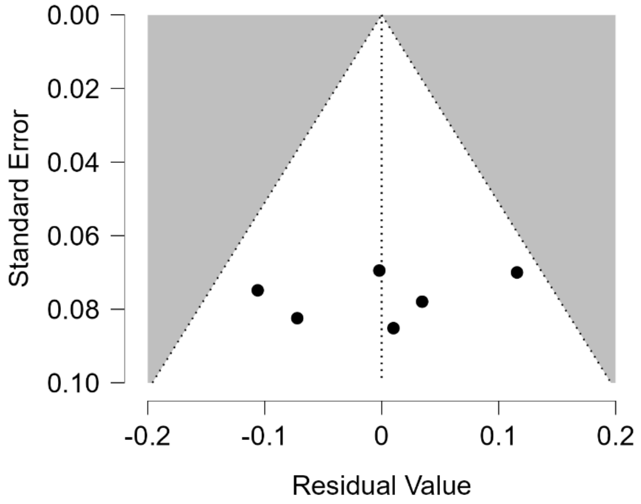

Publication Bias Assessment

Test Name	value	p
Fail-Safe N	1262.000	<.001
Begg and Mazumdar Rank Correlation	-0.067	1.000
Egger's Regression	-0.795	.427
Trim and Fill Number of Studies	0.000	.

Nota. Fail-safe N Calculation Using the Rosenthal Approach

Results of the Meta-analysis on Work Overload and Turnover Intention (JASP and JAMOVI)

Classical Meta-Analysis

Model Summary

Meta-Analytic Tests

	Test	p
Heterogeneity	$Q_e(5) = 10.72$	.057
Pooled effect	$t(5) = 10.65$	< .001

Meta-Analytic Estimates

		95% CI		95% PI	
	Estimate	Lower	Upper	Lower	Upper
Pooled effect	0.2832	0.2173	0.3464	0.1473	0.4085
τ	0.04833	0.000	0.1597		
τ^2	0.002335	0.000	0.02550		
I^2	53.61	0.000	92.66		

Note. The pooled effect is transformed using Fisher's z to r transformation. For nonlinear transformations, the transformed estimate is interpreted as a median on the transformed scale.

Forest Plot

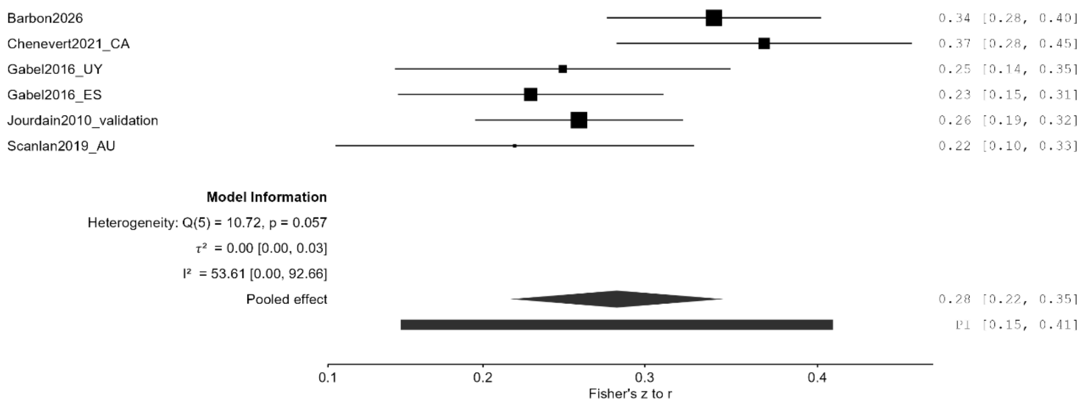

Casewise Diagnostics Table

				Leave One Out				
Standardized Residual	DFFITS	Cook's Distance	Covariance ratio	τ	τ^2	Q_e	Hat	Weight
1.287	0.6556	0.3645	1.112	0.04095	0.001677	6.934	0.2049	20.49
1.797	0.7084	0.3530	0.8034	0.03774	0.001424	6.609	0.1549	15.49
-0.4745	-0.1783	0.03677	1.382	0.05469	0.002992	10.19	0.1348	13.48
-0.9366	-0.4266	0.1865	1.238	0.04830	0.002333	8.561	0.1717	17.17
-0.4062	-0.1789	0.04033	1.515	0.05787	0.003349	9.833	0.2092	20.92
-0.9176	-0.3458	0.1229	1.182	0.04928	0.002428	9.216	0.1245	12.45

Residual Funnel Plot

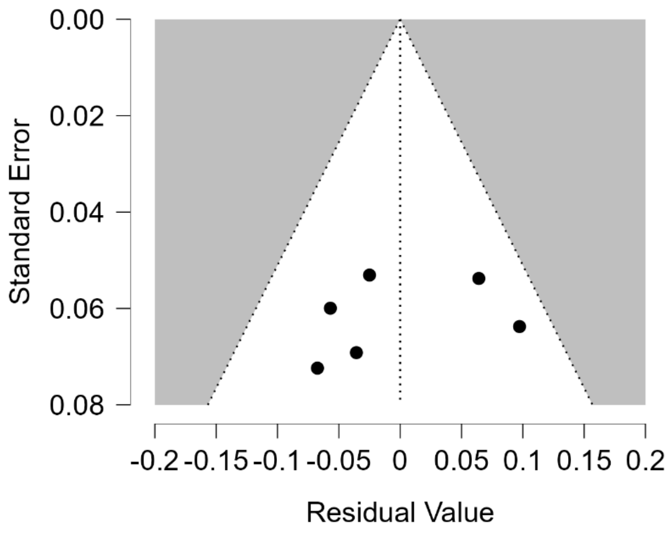

Publication Bias Assessment

Test Name	value	p
Fail-Safe N	548.000	<.001
Begg and Mazumdar Rank Correlation	-0.067	1.000
Egger's Regression	-0.651	.515
Trim and Fill Number of Studies	0.000	.

Nota. Fail-safe N Calculation Using the Rosenthal Approach

TSSEM (MetaSEM R)

Supplementary Note S2. Two-Stage Structural Equation Modeling (TSSEM) Procedure, Uncertainty Propagation, and Reproducibility

1. Methodological Rationale and Uncertainty Propagation Structural meta-analytic mediation was conducted using the Two-Stage Structural Equation Modeling (TSSEM) framework proposed by Cheung and Chan (2005) and Cheung (2015). TSSEM is a two-stage approach designed to pool correlation matrices across primary studies and fit structural equation models to the synthesized matrix:

- Stage 1 (Correlation Pooling): Bivariate random-effects meta-analyses were fitted using Fisher's z-transformation under Restricted Maximum Likelihood (REML) estimation to obtain pooled zero-order correlation coefficients (r) across the triad of variables: Work Overload (WL), Burnout (BO), and Turnover Intention (TI). Each cell in the synthesized correlation matrix (R) represents the back-transformed summary effect size derived from the primary independent samples ($k = 6, N_{total} = 4{,}213$).
- Stage 2 (Structural Model Fitting and Uncertainty Propagation): The hypothesized mediation model (WL → BO → TI, including the direct path WL → TI) was fitted to the synthesized matrix using Weighted Least Squares (WLS) estimation via the metaSEM package (v1.3.0) in R. Uncertainty associated with the pooled correlations was propagated into Stage 2 parameters in two ways:
    1. Harmonic Mean Sample Size (N_{harm}): Following standard TSSEM conventions, the harmonic mean of the sample sizes across the primary studies ($N_{harm} = 1{,}150$) was used as the Stage 2 sample size. Using the harmonic mean provides a conservative effective sample size that heavily penalizes sample size dispersion across studies, preventing larger primary studies from artificially inflating overall statistical power or underestimating standard errors.
    2. Asymptotic Variance-Covariance Matrix: Standard errors (SE), z-statistics, and 95% Wald confidence intervals for the regression paths (a, b, c), as well as for the defined indirect ($a \times b$) and total ($c + a \times b$) effects, were estimated directly from the asymptotic covariance matrix generated by OpenMx/metaSEM. This structure accounts for the sample precision of the pooled correlation entries when deriving inferential statistics for structural coefficients.

2. Complete R Analytical Syntax for Reproducibility

```
# ==============================================================================
# Script: Two-Stage Structural Equation Modeling (TSSEM)
# Purpose: Exploratory Meta-Analytic Mediation Analysis
# Package: metaSEM (v1.3.0) / OpenMx
# ==============================================================================
# Load required package
library(metaSEM)
# Step 1: Define the synthesized meta-analytic correlation matrix (R)
# Variables: WL = Work Overload, BO = Burnout, IT = Turnover Intention
R_matrix <- matrix(
  c(1.0000, 0.5824, 0.2831,  # WL
    0.5824, 1.0000, 0.4117,  # BO
    0.2831, 0.4117, 1.0000), # IT
  nrow = 3, ncol = 3, byrow = TRUE,
  dimnames = list(c("WL", "BO", "IT"), c("WL", "BO", "IT"))
)
```

```
print(R_matrix)

# Step 2: Set the effective sample size using the harmonic mean (N_harm)

# Calculated from primary study samples: N_harm = 1,150

N_harm <- 1150

# Step 3: Specify the structural mediation model using lavaan syntax

model_syntax <- "

  # Direct regression paths

  BO ~ a * WL

  IT ~ b * BO + c * WL

  # Defined parameters (Indirect and Total Effects)

  indirect := a * b

  total    := c + (a * b)

"

# Step 4: Convert lavaan model formulation to RAM specification

ram_model <- lavaan2RAM(model_syntax, obs.variables = c("WL", "BO", "IT"))

# Step 5: Fit Stage 2 WLS structural model

tssem_fit <- wls(Cov = R_matrix, n = N_harm, RAM = ram_model, cor.analysis = TRUE)
```

Step 6: Print model parameter estimates, standard errors, and 95% CIs

summary(tssem_fit)

Result

```
       WL     BO     TI
WL 1.0000 0.5824 0.2831
BO 0.5824 1.0000 0.4117
TI 0.2831 0.4117 1.0000

Call:
wls(Cov = R_matrix, aCov = acov, n = N_harm, RAM = ram_model,
    cor.analysis = TRUE)

95% confidence intervals: z statistic approximation
Coefficients:
    Estimate  Std.Error     lbound     ubound z value Pr(>|z|)
a 0.58240000 0.01948623 0.54420769 0.62059231 29.8878  < 2e-16 ***
b 0.37351504 0.03144937 0.31187539 0.43515468 11.8767  < 2e-16 ***
c 0.06556484 0.03296025 0.00096394 0.13016575  1.9892  0.04668 *
---
Signif. codes:  0 ‘***’ 0.001 ‘**’ 0.01 ‘*’ 0.05 ‘.’ 0.1 ‘ ’ 1

mxAlgebras objects:
indirecto     total
0.2175352 0.2831000

Goodness-of-fit indices:
                                              Value
Sample size                                  1150.0
Chi-square of target model                      0.0
DF of target model                              0.0
p value of target model                         0.0
Number of constraints imposed on "Smatrix"      0.0
DF manually adjusted                            0.0
Chi-square of independence model             1087.9
DF of independence model                        3.0
RMSEA                                           0.0
RMSEA lower 95% CI                              0.0
RMSEA upper 95% CI                              0.0
SRMR                                            0.0
TLI                                            -Inf
CFI                                             1.0
AIC                                             0.0
```
